# 3’-UTR Polymorphisms in the MiRNA Machinery Genes *DROSHA*, *DICER1*, *RAN*, and *XPO5* Are Associated with Colorectal Cancer Risk in a Korean Population

**DOI:** 10.1371/journal.pone.0131125

**Published:** 2015-07-06

**Authors:** Sung Hwan Cho, Jung Jae Ko, Jung Oh Kim, Young Joo Jeon, Jung Ki Yoo, Jisu Oh, Doyeun Oh, Jong Woo Kim, Nam Keun Kim

**Affiliations:** 1 Department of Biomedical Science, College of Life Science, CHA University, Seongnam, South Korea; 2 Institute for Clinical Research, CHA Bundang Medical Center, CHA University, Seongnam, South Korea; 3 Department of Internal Medicine, CHA Bundang Medical Center, CHA University, Seongnam, South Korea; 4 Department of Surgery, CHA Bundang Medical Center, CHA University, Seongnam, South Korea; Dana-Farber Cancer Institute, UNITED STATES

## Abstract

MicroRNAs play an important role in cancer initiation and development. The aim of this study was to investigate whether polymorphisms in miRNA machinery genes are associated with the development of colorectal cancer (CRC). *RAN* rs14035 CT heterozygotes and T allele carriers (CT + TT) genotypes had lower risk of CRC, while the *DICER1* rs3742330, *DROSHA* rs10719, and *XPO5* rs11077 polymorphisms were not associated with CRC in the full study sample. Specifically, male *RAN* rs14035 CT heterozygotes and *XPO5* rs11077 AA genotype (CT/AA) carriers experienced reduced CRC susceptibility (both colon and rectal). Subgroup analysis demonstrated that the combined *RAN* rs14035 CT + TT genotype was associated with rectal cancer, but not colon cancer. In addition, the *DICER1* rs3742330 AG genotype was associated with a significantly increased risk of colon cancer. Stratified analysis revealed the *RAN* rs14035 combined CT+TT genotype was associated with decreased CRC risk in male patients without diabetes mellitus (DM) and in patients with rectal cancer. In addition, we found the *RAN* rs14035 CC genotype was related to a decreased risk of CRC with respect to tumor size and metabolism of homocysteine and folate. Furthermore, patients diagnosed with hypertension or DM who carried the *DROSHA* rs10719 CC genotype showed increased CRC risk, while the *XPO5* rs11077 AC+CC genotype led to increased CRC risk in patients with hypertension only. Our results indicate variations in *RAN *rs14035, *DICER1* rs3742330, *XPO5* rs11077, and *DROSHA* rs10719 of Korean patients are significantly associated with their risk of CRC.

## Introduction

Colorectal cancer (CRC) is the third most common cancer in men (746,000 cases per year, 10.0% of total cancer cases) and the second most common in women (614,000 cases per year, 9.2% of total cancer cases) worldwide [1]. The geographical incidence of CRC varies, but patterns are similar in men and women. Nearly 55% of CRC cases occur in more developed regions [2], although they can arise sporadically.

Several intrinsic factors [e.g., age, sex, diabetes mellitus (DM), obesity, and inflammatory bowel disease] and extrinsic factors (e.g., cigarette smoking, inadequate fiber intake, high alcohol consumption, red meat consumption, and a high-fat diet) are associated with increased CRC risk [3–5]. Thus, CRC susceptibility is influenced by both genetic and environmental elements. Several reports have shown that microRNAs (miRNAs) modulate gene expression by targeting mRNA for deregulation or translational repression [6, 7]. These molecules carry out their biological functions by binding to the 3’-untranslated region (UTR) of target messenger RNA (mRNA), thereby repressing its expression. A single miRNA may regulate multiple targets and thus act as a master control of gene expression. Previous bioinformatics analyses suggest that up to 30% of human genes may be regulated by miRNA, despite the fact that miRNA constitutes only 1–3% of the human genome [8]. Therefore, miRNA appears to play a pivotal role in both physiological and pathological mechanisms [9]. Expression of miRNA is associated with various cancers, and miRNA genes are thought to function as both tumor suppressors and oncogenes [10–12].

MicroRNA machinery proteins, such as DROSHA, RAS-related nuclear protein (RAN), DICER1, and exportin 5 (XPO5), must process miRNAs before they can function. After transcription, DROSHA and its cofactor, DGCR8, process primary miRNA into precursor-miRNA (pre-miRNA) by removing the 5’ cap, the 3’ poly(A) tail, and sequences flanking the hairpin structure. Pre-miRNA is then exported from the nucleus to the cytoplasm by XPO5 and RAN*-*GTP. In the cytoplasm, precursor-miRNA is further processed by DICER1, an endoribonuclease, to produce a short double-stranded RNA fragment (approximately 20–25 nucleotides long) that consists of the mature miRNA and its complementary strand [13]. Due to asymmetric thermostability, the mature miRNA strand is preferentially incorporated into the RNA-induced silencing complex that targets endogenous mRNA for silencing [14].

Recent studies suggest 35% of all CRC cases can be attributed to inherited genetic factors [15]. Inherited risk is likely due to single nucleotide polymorphisms (SNPs) and other genetic abnormalities within coding and noncoding DNA. Because miRNAs play an important role in cancer, including CRC initiation and development, SNPs in miRNA machinery genes may disrupt miRNA structure, binding sites, or processing, thereby altering an individual's susceptibility to CRC by impacting miRNA functionality [16]. For example, impaired miRNA processing may promote cellular transformation and tumorigenesis [17]. In addition, numerous studies have demonstrated an association between the concentration or function of miRNA pathway components and a patient’s prognosis in skin, lung, breast, and ovarian cancer cases [18–21]. Other studies have established that miRNA expression plays a functional role in the initiation and progression of CRC [22,23].

However, to date, few studies have investigated the potential relationship between specific SNPs in miRNA machinery genes and risk of CRC [24–26]. Thus, we investigated if polymorphisms in *DICER1* (rs3742330), *DROSHA* (rs10719), *RAN* (rs14035), and *XPO5* (rs11077) were associated with CRC incidence in a Korean population.

## Materials and Methods

### Study population

Between June 2005 and January 2009, 808 blood samples were collected from the study group, consisting of 408 CRC patients and 400 randomly selected non-CRC (control) subjects, following a health screening at CHA Bundang Medical Center (Seongnam, South Korea). The CRC group included 167 consecutive patients with rectal cancer and 241 consecutive patients with colon cancer. Pathological staging frequencies after curative tumor resection were as follows: tumor node metastasis (TNM) stage I, n = 43 (10.5%); stage II, n = 173 (42.4%); stage III, n = 153 (37.5%); and stage IV, n = 39 (9.6%). Patients with high baseline blood pressure (≥ systolic 140 mmHg or diastolic 90 mmHg) on more than one occasion or with a history of taking antihypertensive medication were classified as having hypertension (HTN). Patients with high fasting plasma glucose (≥ 126 mg/dl), who were taking an oral hypoglycemic agent, or who had a history of insulin treatment were classified as having DM. All study subjects were ethnic Koreans and provided written informed consent for study participation. The study protocol was approved by the Institutional Review Board of CHA Bundang Medical Center.

### Analysis of miRNA biogenesis gene polymorphisms

Genomic DNA was extracted from peripheral blood samples collected with an anticoagulant using a G-DEX blood extraction kit (iNtRON Biotechnology, Seongnam, South Korea). Nucleotide changes were determined via a polymerase chain reaction (PCR)-restriction fragment length polymorphism (RFLP) analysis. Restriction enzyme digestion was carried out using the following enzymes (New England BioLabs, Ipswich, MA): *Nla*III (*DROSHA*, rs10719), *Ban*I (*DICER1*, rs3742330), *Bsl*I (*RAN*, rs14035), and *Bsm*I (*XPO5*, rs11077). Digestion was carried out at 37°C for 16 h.

Genotypes determined by RFLP analysis were confirmed by two independent investigators and by sequencing 10% of the samples. For *RAN* rs14035 in the control group and *XPO5* rs11077 in CRC patients, genotypes were verified three times to rule out possible errors attributed to violation of Hardy-Weinberg equilibrium (HWE).

### Statistical analysis

To compare baseline characteristics between cases and controls, we used Chi-squared tests to analyze categorical data (sex, HTN, and DM) and the Student’s t-test to analyze continuous data. Allele frequencies were calculated to identify deviations from HWE. Adjusted odds ratios (AOR) and 95% confidence intervals (CI) were used to examine the association between *DICER1*, *DROSHA*, *RAN*, *XPO5* polymorphisms and CRC occurrence with GraphPad Prism 4.0 (La Jolla, CA, USA) and MedCalc version 12.1.4 (Panmun Education, Seoul, Korea). Gene interactions among SNP loci were analyzed using multifactor dimensionality reduction (MDR) with MDR version 2.0 (www.epistasis.org) [27–29]. We determined the best multilocus combinations based on MDR identification of the most significant models using maximized cross-validation values. HAPSTAT version 3.0 (www.bios.unc.edu/~lin/hapstat/) was used to estimate haplotype frequencies for the polymorphisms determined by MDR analysis to have strong synergistic effects [30]. The false-positive discovery rate (FDR) correction was used to adjust multiple comparison tests.

## Results

### Study subject characteristics

The 408 CRC cases included 170 males and 238 females with an overall mean age was 61.55±12.26 years (mean±SD; Table 1). Among the cases, 241 (59.1%) had colon cancer and 167 (40.9%) had rectal cancer. No statistically significant differences in age or sex were identified between CRC cases and controls (*P* = 0.772 and 0.847, respectively). However, both HTN (60.5%, *P* = 0.004) and DM (34.1%, *P* < 0.0001) were significantly more common among CRC cases than in controls.

**Table 1:** Baseline characteristics of colorectal cancer patients and control subjects.

Characteristics	Control	CRC	*P* *	Colon	*P* *	Rectum	*P* *
N	400	408		241		167	
Age: years (mean±SD)	61.31±11.63	61.55±12.26	0.772	61.55±12.76	0.802	61.55±11.55	0.823
Gender male: n (%)	172 (43.0)	170 (41.7)	0.847	97 (40.2)	0.707	73 (43.7)	0.933
Hypertension: n (%)	155 (38.8)	247 (60.5)	0.0004	144 (59.8)	0.003	103 (61.7)	0.004
Diabetes mellitus: n (%)	52 (13.0)	139 (34.1)	<0.0001	87 (36.1)	<0.0001	52 (31.1)	<0.0001
Homocysteine: μmol/l (n)	9.84±4.31	10.32±7.56	0.272	10.27±7.95	0.385	10.40±7.00	0.256
Folate: ng/ml (n)	8.68±7.72	7.63±6.61	0.044	7.60±6.50	0.082	7.67±6.79	0.155
Tumor size: n (%)							
<5cm	-	164 (40.2)		84 (34.9)		80 (47.9)	
≥5cm	-	244 (59.8)		157 (65.1)		87 (52.1)	
TNM stage: n (%)							
I	-	43 (10.5)		23 (9.5)		20 (12.0)	
II	-	173 (42.4)		107(44.4)		66 (39.5)	
III	-	153 (37.5)		88 (36.5)		65 (38.9)	
IV	-	39 (9.6)		23 (9.5)		16 (9.6)	

CRC, colorectal cancer; SD, standard deviation; TNM, tumor node metastasis.

* *P*-values were calculated using chi-squared tests for categorical data and two–side t-tests for continuous data.

### Genotype frequencies

Genotype and allele frequencies for the four miRNA machinery genes in CRC cases and controls are shown in Table 2. Genotype distributions in both groups displayed no departure from HWE. *RAN* rs14035 CT heterozygotes had significantly decreased CRC risk relative to wild type homozygotes (AOR = 0.698; 95% CI, 0.511–0.952; *P* = 0.023). Similarly, the combined CT + TT genotype was associated with decreased CRC risk (AOR = 0.690; 95% CI, 0.510–0.934; *P* = 0.016). However, neither association remained statistically significant after controlling for multiple comparisons using the FDR correction. No association between CRC risk and other polymorphisms was found. In addition, we observed no significant relationship between genotype frequencies for the four miRNA machinery genes and CRC patient survival (Table A in S1 File).

**Table 2:** miRNA machinery gene genotype frequencies and AORs in colorectal cancer cases and controls.

	Controls	Case			
Genotypes	(n = 400)	(n = 408)	AOR(95%CI)^a^	*P*	FDR^b^
***DICER* rs3742330**					
AA	145 (36.3)	125 (30.6)	1.000 (reference)		
AG	181 (45.3)	207 (50.7)	1.279 (0.920–1.779)	0.144	0.288
GG	74 (18.4)	76 (18.7)	1.075 (0.701–1.649)	0.741	0.742
Dominant (AA vs. AG + GG)			1.228 (0.901–1.674)	0.194	0.389
Recessive (AA + AG vs. GG)			0.937 (0.643–1.366)	0.737	0.737
HWE-*P*	0.19	0.551			
***DROSHA* rs10719**					
TT	211 (52.8)	224 (54.9)	1.000 (reference)		
TC	168 (42.0)	154 (37.7)	0.841 (0.619–1.143)	0.268	0.358
CC	21 (5.2)	30 (7.4)	1.314 (0.705–2.449)	0.39	0.742
Dominant (TT vs. TC + CC)			0.892 (0.665–1.196)	0.445	0.45
Recessive (TT + TC vs. CC)			1.425 (0.778–2.610)	0.252	0.737
HWE-*P*	0.09	0.62			
***RAN* rs14035**					
CC	233 (58.3)	267 (65.4)	1.000 (reference)		
CT	150 (37.5)	128 (31.4)	0.698 (0.511–0.952)	0.023	0.093
TT	17 (4.3)	13 (3.2)	0.653 (0.295–1.443)	0.292	0.742
Dominant (CC vs. CT + TT)			0.690 (0.510–0.934)	0.016	0.065
Recessive (CC + CT vs. TT)			0.729 (0.333–1.595)	0.429	0.737
HWE-*P*	0.24	0.62			
***XPO5* rs11077**					
AA	337 (84.3)	333 (81.6)	1.000 (reference)		
AC	61 (15.3)	74 (18.1)	1.179 (0.795–1.740)	0.418	0.418
CC	2 (0.4)	1 (0.3)	0.664 (0.058–7.618)	0.742	0.742
Dominant (AA vs. AC + CC)			1.161 (0.788–1.712)	0.45	0.45
Recessive (AA + AC vs. CC)			0.634 (0.055–7.259)	0.714	0.737
HWE-*P*	0.668	0.137			

^a^Adjusted odds ratio on the basis of risk factors, such as age, gender, hypertension, diabetes mellitus.

^b^False positive discovery rate (FDR)-adjusted *P*-value.

In a subgroup analysis targeting cancer type, the combined *RAN* rs14035 CT + TT genotype was associated with decreased risk of rectal cancer (AOR = 0.640; 95% CI, 0.430–0.954; *P* = 0.028), but not colon cancer (Table 3). Conversely, *DICER1* rs3742330 AG heterozygotes had a significantly increased risk of colon but not rectal cancer (AOR = 1.506; 95% CI, 1.020–2.223; *P* = 0.040). However, the relationship was not significant after FDR correction, suggesting a weak association (Table 3). Combination analyses (Table 4 & Table B in S1 File) revealed that *RAN* rs14035 CT heterozygotes and *XPO5* rs11077 AA carriers experienced reduced susceptibility of CRC (AOR = 0.610; 95% CI, 0.434–0.859; *P* = 0.005) (Table 4). Moreover, these same genotypes were associated with reduced susceptibility of both cancer types in male (AOR = 0.283; 95% CI, 0.139–0.573; *P* = 0.001, AOR = 0.497; 95% CI, 0.254–0.973; *P* = 0.041; Table 5 & Table C in S1 File), but not female patients (Table D in S1 File). Although *DROSHA* rs10719 CC genotype carriers and *RAN* rs14035 CC heterozygotes showed reduced risk of rectal cancer (Table E in S1 File), we observed no significant association between genotype frequencies for the four miRNA machinery genes and patient survival or TNM classification stage of their malignant CRC tumors (Table F in S1 File and Table G in S1 File).

**Table 3:** miRNA machinery gene genotype frequencies and AORs in colon and rectal cancer cases and controls.

	Controls	Colon				Rectum			
Genotypes	(n = 400)	(n = 241)	AOR(95% CI)^a^	*P*	FDR^b^	(n = 167)	AOR(95% CI)^a^	*P*	FDR^b^
***DICER* rs3742330**									
AA	145 (36.3)	67 (27.8)	1.000 (reference)			58 (34.7)	1.000 (reference)		
AG	181 (45.3)	130 (53.9)	1.506 (1.020–2.223)	0.04	0.152	77 (46.1)	0.993 (0.649–1.519)	0.974	0.984
GG	74 (18.4)	44 (18.3)	1.143 (0.686–1.904)	0.608	0.633	32 (19.2)	0.952 (0.552–1.641)	0.858	0.984
Dominant (AA vs. AG + GG)			1.398 (0.966–2.024)	0.076	0.152		1.004 (0.676–1.490)	0.984	0.984
Recessive (AA + AG vs. GG)			0.899 (0.580–1.393)	0.633	0.633		0.974 (0.600–1.581)	0.917	0.984
HWE-*P*	0.19	0.168				0.478			
***DROSHA* rs10719**									
TT	211 (52.8)	136 (56.4)	1.000 (reference)			88 (52.7)	1.000 (reference)		
TC	168 (42.0)	90 (37.3)	0.835 (0.584–1.194)	0.324	0.795	64 (38.3)	0.841 (0.564–1.254)	0.395	0.527
CC	21 (5.2)	15 (6.2)	1.105 (0.520–2.347)	0.795	0.795	15 (9.0)	1.570 (0.750–3.288)	0.231	0.462
Dominant (TT vs. TC + CC)			0.865 (0.614–1.218)	0.407	0.795		0.916 (0.627–1.340)	0.652	0.652
Recessive (TT + TC vs. CC)			1.211 (0.588–2.498)	0.604	0.795		1.677 (0.814–3.455)	0.161	0.462
HWE-*P*	0.09	0.983				0.498			
***RAN* rs14035**									
CC	233 (58.3)	154 (63.9)	1.000 (reference)			113 (67.7)	1.000 (reference)		
CT	150 (37.5)	76 (31.5)	0.712 (0.495–1.025)	0.068	0.17	52 (31.1)	0.679 (0.453–1.019)	0.062	0.116
TT	17 (4.3)	11 (4.6)	0.997 (0.431–2.303)	0.994	0.994	2 (1.2)	0.267 (0.059–1.212)	0.087	0.116
Dominant (CC vs. CT + TT)			0.734 (0.517–1.043)	0.085	0.17		0.640 (0.430–0.954)	0.028	0.112
Recessive (CC + CT vs. TT)			1.085 (0.475–2.478)	0.846	0.994		0.309 (0.069–1.389)	0.126	0.126
HWE-*P*	0.24	0.68				0.135			
***XPO5* rs11077**									
AA	337 (84.3)	195 (80.9)	1.000 (reference)			138 (82.6)	1.000 (reference)		
AC	61 (15.3)	45 (18.7)	1.234 (0.787–1.934)	0.36	0.72	29 (17.4)	1.101 (0.665–1.825)	0.708	0.993
CC	2 (0.4)	1 (0.4)	1.219 (0.105–14.154)	0.874	0.902	0 (0.0)	N/A	0.993	0.993
Dominant (AA vs. AC + CC)			1.235 (0.792–1.926)	0.352	0.72		1.070 (0.647–1.770)	0.791	0.993
Recessive (AA + AC vs. CC)			1.166 (0.101–13.419)	0.902	0.902		N/A	0.993	0.993
HWE-*P*	0.668	0.344				0.219			

^a^Adjusted odds ratio on the basis of risk factors, such as age, gender, hypertension, diabetes mellitus.

^b^False positive discovery rate (FDR)-adjusted *P*-value

**Table 4:** The combination of miRNA machinery genes polymorphisms based on MDR and CRC patients.

	Control	Case						
Genotypes	(n = 400)	(n = 408)	COR(95% CI)	*P*	FDR^b^	AOR(95% CI)^a^	*P*	FDR^b^
***RAN/XPO5***								
CC/AA	191 (47.8)	223 (54.7)	1.000 (reference)			1.000 (reference)		
CC/AC	41 (10.3)	43 (10.5)	0.898 (0.562–1.436)	0.654	0.785	0.845 (0.516–1.384)	0.503	0.848
CC/CC	1 (0.3)	1 (0.2)	0.857 (0.053–13.787)	0.913	0.913	0.799 (0.049–13.104)	0.875	0.952
CT/AA	132 (33.0)	102 (25.0)	0.662 (0.479–0.914)	0.012	0.072	0.610 (0.434–0.859)	0.005	0.03
CT/AC	17 (4.3)	26 (6.4)	1.310 (0.690–2.487)	0.409	0.785	1.219 (0.621–2.389)	0.565	0.848
CT/CC	1 (0.3)	0 (0.0)	NA	NA	NA	NA	NA	NA
TT/AA	14 (3.5)	8 (2.0)	0.489 (0.201–1.192)	0.116	0.348	0.520 (0.203–1.329)	0.172	0.516
TT/AC	3 (0.8)	5 (1.2)	1.428 (0.337–6.051)	0.629	0.785	1.048 (0.224–4.915)	0.952	0.952
TT/CC	0 (0.0)	0 (0.0)	NA	NA	NA	NA	NA	NA

^a^Adjusted odds ratio on the basis of risk factors, such as age, gender, hypertension, diabetes mellitus. MDR, multifactor dimensional reduction.

^b^False positive discovery rate (FDR)-adjusted *P*-value.

**Table 5:** The combination of the polymorphisms of microRNA machinery genes in CRC patients and controls: male subgroup.

	Control	Colon				Rectum			
Characteristics	(n = 172)	(n = 97)	AOR(95% CI)^a^	*P*	FDR^b^	(n = 73)	AOR(95% CI)^a^	*P*	FDR^b^
***DROSHA/RAN***									
TT/CC	57 (33.1)	41 (42.3)	1.000 (reference)			23 (31.5)	1.000 (reference)		
TT/CT	41 (23.8)	15 (15.5)	0.412 (0.181–0.940)	0.035	0.21	17 (23.3)	0.934 (0.434–2.007)	0.861	0.861
TT/TT	5 (2.9)	2 (2.1)	0.613 (0.079–4.769)	0.64	0.878	0 (0.0)	NA	NA	NA
TC/CC	34 (19.8)	25 (25.8)	1.127 (0.541–2.346)	0.75	0.878	21 (28.8)	1.391 (0.646–2.996)	0.4	0.5
TC/CT	26 (15.1)	8 (8.2)	0.409 (0.142–1.176)	0.097	0.291	7 (9.6)	0.563 (0.196–1.615)	0.285	0.5
TC/TT	1 (0.6)	1 (1.0)	0.605 (0.001–368.948)	0.878	0.878	0 (0.0)	NA	NA	NA
CC/CC	3 (1.7)	3 (3.1)	2.768 (0.489–15.649)	0.25	0.5	4 (5.5)	2.549 (0.451–14.413)	0.29	0.5
CC/CT	5 (2.9)	0 (0.0)	NA	NA	NA	1 (1.4)	0.340 (0.032–3.659)	0.373	0.5
CC/TT	0 (0.0)	2 (2.1)	NA	NA	NA	0 (0.0)	NA	NA	NA
***RAN/XPO5***									
CC/AA	74 (43.0)	56 (57.7)	1.000 (reference)			40 (54.8)	1.000 (reference)		
CC/AC	19 (11.0)	13 (13.4)	0.733 (0.302–1.777)	0.492	0.577	8 (11.0)	0.742 (0.284–1.941)	0.543	0.815
CC/CC	1 (0.6)	0 (0.0)	NA	NA	NA	0 (0.0)	NA	NA	NA
CT/AA	63 (36.6)	16 (16.5)	0.283 (0.139–0.573)	0.001	0.005	20 (27.4)	0.497 (0.254–0.973)	0.041	0.123
CT/AC	8 (4.7)	7 (7.2)	0.701 (0.202–2.439)	0.577	0.577	5 (6.8)	0.940 (0.267–3.308)	0.923	0.923
CT/CC	1 (0.6)	0 (0.0)	NA	NA	NA	0 (0.0)	NA	NA	NA
TT/AA	5 (2.9)	1 (1.0)	0.281 (0.026–3.039)	0.296	0.577	0 (0.0)	NA	NA	NA
TT/AC	1 (0.6)	4 (4.1)	2.213 (0.199–24.605)	0.518	0.577	0 (0.0)	NA	NA	NA
TT/CC	0 (0.0)	0 (0.0)	NA	NA	NA	0 (0.0)	NA	NA	NA

^a^Adjusted odds ratio on the basis of risk factors, such as age, gender, hypertension, diabetes mellitus.

^b^False positive discovery rate (FDR)-adjusted *P*-value.

Stratified analyses indicated the *RAN* rs14035 combined CT+TT genotype was associated with decreased CRC risk in male patients (AOR = 0.493; 95% CI, 0.308–0.791; *P* = 0.003), patients without DM (AOR = 0.618; 95% CI, 0.438–0.874; *P* = 0.006), and patients with rectal cancer (AOR = 0.640; 95% CI, 0.43–0.954; *P* = 0.028; Table 6). In addition, we determined the *DROSHA* rs10719 CC genotype was associated with increased risk of colon cancer in subjects at 62 years or older (AOR = 3.148; 95% CI, 1.276–7.766; P = 0.013) and subjects younger than 62 years (AOR = 2.940; 95% CI, 1.169–7.399; P = 0.022; Table 6). Furthermore, the *RAN* rs14035 CC genotype was linked with decreased risk of CRC in subjects with < 5-cm tumors (AOR = 0.654; 95% CI, 0.437–0.978; *P* = 0.039), homocysteine levels lower than 12.97 μmol/l (AOR = 0.637; 95% CI, 0.453–0.896; *P* = 0.01), and folate levels higher than 3.72 ng/ml (AOR = 0.630; 95% CI, 0.447–0.888; *P* = 0.008) (Table 7). We also found the *DROSHA* rs10719 CC genotype was associated with increased CRC risk in subjects with < 5-cm tumors (AOR = 2.159; 95% CI, 1.057–4.413; *P* = 0.035).

**Table 6:** Stratified effect of miRNA machinery gene polymorphisms on colorectal cancer risk (I).

Factor	*DICER* rs3742330 AG+GG		*DROSHA* rs10719 CC		*RAN* rs14035 CT+TT		*XPO5* rs11077 AC+CC	
	AOR (95% CI)^a^	*P*	AOR (95% CI)^a^	*P*	AOR (95% CI)^a^	*P*	AOR (95% CI)^a^	*P*
Age								
<62years	1.350 (0.844–2.159)	0.210	2.940 (1.169–7.399)	0.022	0.749 (0.480–1.167)	0.201	1.166 (0.662–2.055)	0.594
≥62years	1.490 (0.955–2.324)	0.079	3.148 (1.276–7.766)	0.013	0.718 (0.469–1.101)	0.129	0.955 (0.560–1.630)	0.867
Gender								
Male	1.296 (0.814–2.064)	0.275	1.172 (0.431–3.192)	0.756	0.493 (0.308–0.791)	0.003	1.184 (0.673–2.085)	0.558
Female	1.178 (0.776–1.790)	0.442	1.622 (0.752–3.503)	0.218	0.872 (0.585–1.299)	0.500	1.111 (0.647–1.909)	0.702
Hypertension								
No	1.385 (0.889–2.158)	0.150	1.793 (0.757–4.248)	0.184	0.732 (0.469–1.141)	0.168	1.093 (0.616–1.936)	0.762
Yes	1.008 (0.644–1.579)	0.971	1.246 (0.537–2.895)	0.609	0.696 (0.458–1.057)	0.089	1.238 (0.718–2.135)	0.442
Diabetes mellitus								
No	1.25 (0.878–1.767)	0.219	1.300 (0.660–2.561)	0.448	0.618 (0.438–0.874)	0.006	1.195 (0.768–1.861)	0.430
Yes	1.115 (0.557–2.229)	0.759	2.039 (0.434–9.592)	0.367	1.006 (0.520–1.946)	0.987	1.001 (0.443–2.260)	0.998
Tumor site								
Colon	1.398 (0.966–2.024)	0.076	1.211 (0.588–2.498)	0.604	0.734 (0.517–1.043)	0.085	1.235 (0.792–1.926)	0.352
Rectum	1.004 (0.676–1.490)	0.984	1.677 (0.814–3.455)	0.161	0.640 (0.430–0.954)	0.028	1.070 (0.647–1.770)	0.791

^a^Adjusted odds ratio on the basis of risk factors, such as age, gender, hypertension, diabetes mellitus.

**Table 7:** Stratified effect of miRNA machinery gene polymorphisms on colorectal cancer risk (II).

Factor	*DICER* rs3742330A>G		*DROSHA* rs10719T>C		*RAN* rs14035 C>T		*XPO5 rs11077* A>C	
	AOR (95% CI)^a^	*P*	AOR (95% CI)^a^	*P*	AOR (95% CI)^a^	*P*	AOR (95% CI)^a^	*P*
Tumor size								
<5cm	1.100 (0.735–1.646)	0.642	2.159 (1.057–4.413)	0.035	0.654 (0.437–0.978)	0.039	1.031 (0.616–1.724)	0.909
≥5cm	1.283 (0.892–1.846)	0.180	0.958 (0.460–1.997)	0.909	0.732 (0.517–1.038)	0.08	1.255 (0.809–1.948)	0.311
Lymph node invasion								
No	1.136 (0.785–1.642)	0.499	1.503 (0.739–3.058)	0.261	0.736 (0.513–1.057)	0.097	1.153 (0.727–1.830)	0.545
Yes	1.312 (0.884–1.946)	0.177	1.306 (0.626–2.726)	0.477	0.663 (0.453–0.968)	0.034	1.180 (0.733–1.898)	0.496
Homocysteine								
<12.97μmol/l	1.141 (0.807–1.615)	0.456	1.579 (0.821–3.038)	0.171	0.637 (0.453–0.896)	0.01	1.108 (0.710–1.728)	0.652
≥12.97μmol/l	1.717 (0.745–3.958)	0.205	0.504 (0.066–3.877)	0.511	1.013 (0.462–2.221)	0.974	0.881 (0.341–2.277)	0.794
Folate								
<3.72ng/ml	0.858 (0.326–2.261)	0.757	0.384 (0.049–3.010)	0.362	1.046 (0.427–2.565)	0.921	0.964 (0.294–3.153)	0.951
≥3.72ng/ml	1.230 (0.869–1.740)	0.242	1.735 (0.890–3.383)	0.106	0.630 (0.447–0.888)	0.008	1.096 (0.708–1.697)	0.682

^a^Adjusted odds ratio on the basis of risk factors, such as age, gender, hypertension, diabetes mellitus.

Interaction models suggested by MDR, based on a cross-validation value of 10, were evaluated using haplotype-based analysis. However, no associations between the four miRNA machinery polymorphisms and CRC risk were identified (Table H in S1 File and Table I in S1 File).

### Genetic association of miRNA machinery genes and combined gene–patient characteristics

**Fig 1:**
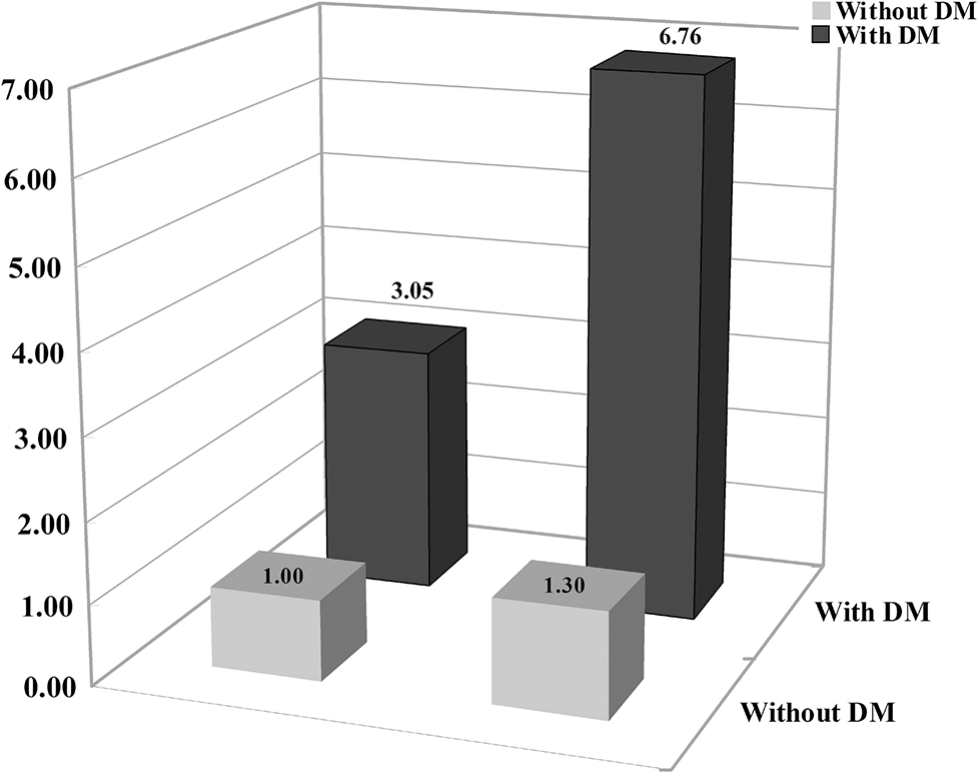
The effects of *DROSHA* rs10719T>C variant on colorectal cancer (CRC) development modulated by diabetes mellitus (DM). Y-axis represents fold changes between *DROSHA* genotype TT+TC and *DROSHA* genotype CC. Each row represents the patients with or without DM. Each column represents *DROSHA* genotype TT+TC and *DROSHA* genotype CC.

We examined the potential genetic association between HTN or DM and gene/patient characteristics to elucidate the genetic etiology of CRC development because metabolic syndrome risk factors, including HTN and DM, are very relevant in the occurrence of CRC. We determined the *DROSHA* rs10719 CC genotype was associated increased risk of colon cancer in subjects 62 years or older (AOR = 3.875; 95% CI, 1.432–10.490) and patients with HTN (AOR, 3.292; 95% CI, 1.362–7.958), DM (AOR = 6.764; 95% CI, 1.424–32.126) (Table 8). Interestingly, the combination of DM and the *DROSHA* rs10719 CC genotype increased CRC risk 6.764-fold (Fig 1). We also observed an association between the *XPO5* rs11077 combined AC+CC genotype and increased CRC risk in patients with HTN (AOR, 3.126; 95% CI, 1.739–5.619) or BMI of < 25 kg/m^2^ (AOR = 11.765; 95% CI, 1.011–3.079) (Table 8).

**Table 8:** Interplay between genes and patients characteristics in shaping colorectal cancer risk.

Factor	*DICER* rs3742330	*DICER* rs3742330	*DROSHA* rs10719	*DROSHA* rs10719	*RAN* rs14035	*RAN* rs14035	*XPO5* rs11077	*XPO5* rs11077
	AA	AG+GG	TT+TC	CC	CC	CT+TT	AA	AC+CC
	AOR (95% CI)^a^	AOR (95% CI)^a^	AOR (95% CI)^a^	AOR (95% CI)^a^	AOR (95% CI)^a^	AOR (95% CI)^a^	AOR (95% CI)^a^	AOR (95% CI)^a^
Age								
<62years	1.000 (reference)	0.971 (0.627–1.503)	1.000 (reference)	0.632 (0.247–1.617)	1.000 (reference)	0.671 (0.434–1.037)	1.000 (reference)	1.472 (0.824–2.630)
≥62years	0.581 (0.251–1.345)	1.209 (0.571–2.562)	1.084 (0.648–1.814)	3.875 (1.432–10.490)	1.020 (0.541–1.920)	0.744 (0.363–1.527)	1.415 (0.820–2.441)	1.562 (0.748–3.260)
Gender								
Male	1.000 (reference)	1.296 (0.814–2.064)	1.000 (reference)	1.172 (0.431–3.192)	1.000 (reference)	0.493 (0.308–0.791)	1.000 (reference)	1.184 (0.673–2.085)
Female	1.129 (0.673–1.894)	1.289 (0.831–2.000)	1.040 (0.766–1.412)	1.631 (0.751–3.541)	0.859 (0.591–1.250)	0.720 (0.461–1.125)	1.084 (0.782–1.502)	1.204 (0.692–2.096)
Hypertension								
No	1.000 (reference)	1.385 (0.889–2.158)	1.000 (reference)	1.793 (0.757–4.248)	1.000 (reference)	0.732 (0.469–1.141)	1.000 (reference)	1.093 (0.616–1.936)
Yes	2.927 (1.709–5.011)	2.726 (1.726–4.305)	2.555 (1.841–3.547)	3.292 (1.362–7.958)	2.543 (1.693–3.820)	1.608 (1.022–2.532)	2.345 (1.656–3.320)	3.126 (1.739–5.619)
DM								
No	1.000 (reference)	1.245 (0.878–1.767)	1.000 (reference)	1.300 (0.660–2.561)	1.000 (reference)	0.618 (0.438–0.874)	1.000 (reference)	1.195 (0.768–1.861)
Yes	3.321 (1.727–6.385)	3.732 (2.269–6.138)	3.054 (2.095–4.452)	6.764 (1.424–32.126)	2.562 (1.608–4.083)	2.535 (1.429–4.497)	3.189 (2.124–4.789)	3.422 (1.583–7.396)
Homocysteine								
<12.97μmol/l	1.000 (reference)	1.141 (0.807–1.615)	1.000 (reference)	1.579 (0.821–3.038)	1.000 (reference)	0.637 (0.453–0.896)	1.000 (reference)	1.108 (0.710–1.728)
≥12.97μmol/l	1.169 (0.555–2.464)	1.813 (1.021–3.220)	1.507 (0.975–2.331)	0.682 (0.091–5.110)	1.169 (0.680–2.008)	1.093 (0.568–2.106)	1.522 (0.947–2.446)	1.200 (0.498–2.892)
Folate								
≥3.72ng/ml	1.000 (reference)	1.230 (0.869–1.740)	1.000 (reference)	1.735 (0.890–3.383)	1.000 (reference)	0.630 (0.447–0.888)	1.000 (reference)	1.096 (0.708–1.697)
<3.72ng/ml	4.185 (1.717–10.201)	3.394 (1.905–6.049)	3.365 (2.092–5.413)	0.941 (0.150–5.919)	2.397 (1.351–4.255)	2.606 (1.250–5.435)	3.191 (1.931–5.273)	2.879 (0.954–8.685)
BMI								
<25kg/m2	1.000 (reference)	0.953 (0.629–1.445)	1.000 (reference)	2.541 (0.975–6.623)	1.000 (reference)	0.703 (0.469–1.054)	1.000 (reference)	1.765 (1.011–3.079)
≥25kg/m2	0.302 (0.159–0.575)	0.684 (0.416–1.127)	0.583 (0.408–0.834)	0.681 (0.219–2.113)	0.517 (0.336–0.794)	0.475 (0.276–0.816)	0.642 (0.442–0.933)	0.535 (0.255–1.121)

^a^Adjusted odds ratio on the basis of risk factors, such as age, gender, hypertension, diabetes mellitus.

## Discussion

Previous studies have shown that several types of cancer are associated with alterations to miRNA machinery genes, such as *DROSHA*, *DICER1*, *XPO5*, and *AGO2* [31–33]. Although modifications to these genes can significantly affect initiation and progression of cancer, the role of genetic variation in miRNA machinery genes during CRC development is not fully understood. To the best of our knowledge, this is the first report to evaluate the association between *RAN* polymorphisms and CRC; we found *RAN* rs14035 CT heterozygotes and T allele carriers (CT + TT genotypes) had a lower CRC risk than individuals with other genotypes.

In addition, in a subgroup analysis targeting cancer type, we determined the combined *RAN* rs14035 CT + TT genotype was associated with decreased rectal cancer risk. RAN encodes a small G protein essential for the translocation of RNA and proteins through the nuclear pore complex [34]. When RAN-GTP is depleted as a result of RAN guanine nucleotide exchange factor inhibition, pre-miRNA export is greatly reduced, indicating miRNA transport is mediated by a RAN-GTP-binding export receptor [35]. Therefore, it is possible that *RAN* mutations play an essential role in pathology-related changes to miRNA transport and expression. The RAN protein is also a well-known downstream modulator of the PI3K signaling pathway, which mediates cancer cell invasion and metastasis [36]. Moreover, CRC tissues exhibit significantly higher levels of *RAN* expression than normal colorectal epithelial cells, which have been positively associated with depth of invasion, lymph node metastases, distant metastases, tumor differentiation, and tumor–node–metastasis stage [37]. Therefore, the rs14035 polymorphism in *RAN*’s 3’-UTR may influence its function as a downstream modulator of CRC development.

Faggad et al. [38] reported that reduced *DICER1* expression may contribute to tumor progression in CRC. Consistent with this report, Dewi et al. [39] determined that *DICER1*-deficient CRC tissues have a reduced number of alkaline phosphatase-positive reprogrammed cells relative to wild type cells. However, another report showed no significant difference in expression levels of long 3'-UTR *DICER1* mRNA between CRC tumors and normal tissues [40]. Thus, the effects of up- and down regulation of *DICER1* expression on CRC susceptibility are unclear. Our subgroup analysis, which target specific cancer type, revealed that *DICER1* rs3742330 AG heterozygotes showed a significantly increased risk of colon cancer. Similarly, an association between this polymorphism, located in the gene’s 3’-UTR, and T-cell lymphoma survival has also been reported, perhaps because its 3′-UTR is important for mRNA transcript stability [41]. Moreover, another SNP located in *DICER1*’s 3'-UTR (rs1057035) may contribute to oral cancer risk by affecting miRNA binding to *DICER1* [42]. Although the *DICER1* rs3742330 AG genotype was associated with a significantly increased risk of colon cancer in our data, we observed no significant relationship between the genotype frequencies for the *DICER1* rs3742330 and CRC patient survival. *DICER1*rs3742330 was reported to be associated with increased survival in T-cell lymphoma [41]. These inconsistent results across different cancers suggest that the abnormal expression patterns of miRNA pathway genes might be associated with tissue-specific effects. The SNP is located in the 3’-UTR, a region that is important for *DICER1* mRNA stability, polyadenylation, translation efficiency, and localization. The 3'-UTR contains binding sites for microRNAs. Many studies have reported the regulatory roles of miRNAs in genetic networks underlying various cellular pathways, indicating that oncogenic miRNAs might be involved in the genetic networks regulating the functional pathway deregulated in different cancer cells [7,43]. *DICER1* rs3742330 has been identified as the target site of two miRNAs—miR-3622a-5p [44] and miR-5582-5p [45]. However, the regulation of these miRNAs of *DICER1* rs3742330 in CRC and T-cell lymphoma has not been experimentally validated. The expression levels of *DICER1* have global effects on the biogenesis of miRNA. For instance, a pattern of down-regulation of *DICER1* is associated with poor prognosis in skin, lung, breast, and ovarian cancers, among other cancers [46]. However, the analysis of prostate cancer and CRC shows overexpression of *DICER1* and other miRNA biogenesis genes in metastatic lesions [47]. Mucoepidermoid cancers arising in the throat or upper esophagus exhibit both over- and under-expression of *DICER1* compared with normal tissues from the throat and esophagus [48]. The miRNA-SNP of rs3742330 of *DICER1* has been identified for its association with the cancer outcome of CRC, T-cell lymphoma, oral premalignant lesions and renal cell carcinoma [49–51,42].The AA allele of rs3742330 located in the *DICER1* gene exhibited a significantly increased risk of CRC. However, in T-cell lymphoma, Patients carrying the GG genotype had a significantly increased overall survival (OS) compared with those carrying the GA and AA genotypes. In oral premalignant lesions, patients carrying the GA and AA genotypes had a significantly increased risk of OPL, and there was no significant relationship between the genotype frequencies for the *DICER1* rs3742330 and renal cell carcinoma. These studies, together with our study, suggest genetic polymorphisms in cancer may play a different role in the regulation of miRNA machinery genes, including *DICER1*, and therefore influence the prognosis of cancer.

In the miRNA processing system, the XPO5/RAN-GTP complex mediates the nuclear transport of pre-miRNAs. *XPO5* mutants display reduced miRNA processing levels and target inhibition, while restored XPO5 acts as a tumor suppressor that reverses the impaired export of pre-miRNA in colon cancer [52]. Previous studies have reported associations between the *XPO5* rs11077 SNP (located in the gene’s 3’UTR) and esophageal cancer, non-small cell lung cancer, and multiple myeloma [53,54]. In addition, associations between polymorphisms in the *XPO5* and *AGO1* genes and renal cell carcinoma risk have been reported [55]. In particular, *XPO5* rs11077 was linked with increased risk of renal cell carcinoma in a recessive model [55]. In support of these earlier reports, we found that *RAN* rs14035 CT heterozygotes and *XPO5* rs11077 AA genotype carriers experienced reduced susceptibility to CRC, a link that was especially apparent in male patients. Gender differences have been documented among patients diagnosed with CRC. A higher CRC age-adjusted incidence among men than among women has persisted over the past 30 years, but the underlying cause remains unclear [56]. In our study, *RAN* rs14035 CT/*XPO5* rs11077 AA and *RAN* rs14035 CT/*DROSHA* rs10719 AA genotypes were associated with reduced susceptibility to CRC in males. In addition, *RAN* rs14035 CT heterozygotes and *DROSHA* rs10719 AA genotype carriers reduced susceptibility to CRC subtype. Polymorphisms within the 3'-UTRs of miRNA machinery genes may be responsible for locally altered mRNA secondary structures. For example, 3’-UTR polymorphisms can result in different secondary mRNA structures and distinct allele-dependent differences in mRNA stability [57]. Similarly, several reports have suggested that SNPs outside of the miRNA binding site result in altered miRNA binding due to allele-dependent changes in secondary mRNA structure [58]. In addition, alterations in secondary structure can interfere with RNA-binding proteins, which can lead to altered mRNA stability [59]. Thus, the SNPs located in the 3’-UTRs of *RAN*, *DICER1* and *XPO5* may affect mRNA stability and subsequent expression. However, the results from this study require validation by another CRC case-control study and by laboratory-based expression methods.

Recent evidence shows that components of metabolic syndrome (MetS), including aging, HTN, and DM, may also be associated with the risk of developing CRC [60,61]. The relationship between individual components of MetS and CRC risk has been analyzed by several studies [62,63]. But an inconsistent link between MetS and its components on CRC mortality was observed in another study [64]. Diabetes mellitus type 2 is associated with a 20%-60% increased risk of CRC [65], as insulin resistance may promote carcinogenesis directly by stimulating colonic cell growth [66]. Our data show that the *RAN* rs14035 CT + TT genotype may result in lower CRC risk in patients without DM, but not in patients with DM. However, a recent meta-analysis demonstrated that DM was an independent, increased risk factor for CRC in both men and women, even after controlling for smoking, obesity, and physical exercise of the patients [65]. However, we found the *RAN* rs14035 CT + TT genotype was associated with a lower CRC risk in male patients and in all patients with rectal cancer. Although numerous studies have shown that tumor size is of no prognostic significance in CRC [67,68], our data showed that *DROSHA* rs10719 CC was associated with an increased CRC risk regarding tumor size (<5 cm) and age (≥62 years). We also found that the *DICER1* rs3742330 and *XPO5* rs11077 genotypes were not associated with the risk of age, gender, hypertension, DM, tumor site, tumor size, lymph node invasion, HTN and folate, suggesting that these SNPs might not modulate the susceptibility to CRC in the Korean population.

Several studies have reported the associations between homocysteine levels and cancer development. For example, cohort studies found elevated homocysteine was correlated with increased adenoma recurrence [69,70]. Similarly, we observed that *DROSHA* rs10719 and *RAN* rs14035 CC genotypes were associated with CRC with respect to the subjects’ tumor sizes. We also found that the *RAN* rs14035 CC genotype was associated with decreased risk of CRC in patients with < 12.97 μmol/l homocysteine. However, to our knowledge, no other significant associations have been found between homocysteine and risk of CRC [71].

Folate provides 1-carbon units for DNA synthesis and methylation, and neoplasia results from the disruption in both of these processes. Modifications to DNA methylation occur on a genomic- and gene-specific level in colorectal neoplasia [72], and some studies have shown that low plasma folate concentrations were associated with reduced CRC risk and that plasma folate concentrations were positively related to CRC risk [73,74]. However, our data show that the *RAN* rs14035 CC genotype was associated with decreased risk of CRC in subjects with > 3.72 ng/ml folate.

We also examined the interplay between genetic factors and patient characteristics with respect to CRC occurrence. For example, we determined the *DROSHA* rs10719 CC genotype was associated with increased CRC risk in patients with HTN and DM, while the *XPO5* rs11077 AC+CC genotype correlated to increased CRC risk in patients with HTN. Recent studies have observed increased risk of CRC associated with HTN [60,62], and the presence of HTN, obesity, and hyperglycemia [75]. However, a study of Finnish male smokers did not support these associations [76]. In addition, our data showed that the CC genotype of rs10719 located in the *DROSHA* gene had an increased CRC risk in patients with age (≥62 years) compared with those carrying the TT and CC genotypes. An accumulation of evidence indicates MetS, with its systemic and hormonal effects, may affect a patient’s susceptibility to carcinoma and the prognosis of patients diagnosed with various cancers [77,78]. Our study provides new information regarding the association between CRC and MetS in the context of polymorphisms in miRNA machinery genes. However, additional studies of other miRNA machinery genes will be needed to clarify the association between these polymorphisms and CRC.

In summary, we investigated the relationship between CRC susceptibility and the miRNA machinery gene *DROSHA* rs10719, *DICER1* rs3742330, *RAN* rs14035, and *XPO5* rs11077 polymorphisms. To date, few studies have investigated the association between polymorphisms in *RAN* gene and CRC risk and there were no associations between the *RAN* gene polymorphisms and CRC risk [24–26]. Though our results provide the first evidence for a significant association between *RAN* polymorphisms and CRC in Korean patients, our findings will benefit from additional data regarding the polymorphism’s effect upon mRNA stability, binding efficiency, and selectivity. Therefore, our study, while novel in its findings, requires validation by functional studies investigating the polymorphism’s effect upon miRNA machinery and downstream cellular activities.

## Supporting Information

S1 File Table A in S1 File. miRNA biogenesis gene genotype frequencies and CRC patient survival. Table B in S1 File. The combination of miRNA biogenesis genes polymorphisms based on MDR and CRC patients. Table C in S1 File. The combination of the polymorphisms of microRNA machinery genes in CRC patients and controls: male subgroup. Table D in S1 File. The combination of the polymorphisms of microRNA machinery genes in CRC patients and controls: female subgroup. Table E in S1 File. The combination of the polymorphisms of microRNA machinery genes between the subgroup for colorectal cancer and control subjects. Table F in S1 File. Genotype frequencies of miRNA biogenesis gene polymorphisms and CRC patients survival in TNM stage I + II. Table G in S1 File. Genotype frequencies of miRNA biogenesis genes polymorphisms and CRC patients survival in TNM stage III + IV. Table H in S1 File. Allele combinations of miRNA processing genes in CRC patients and controls using multifactor dimensionality reduction. Table I in S1 File. Allele combination of miRNA biogenesis genes polymorphisms based on multifactor dimensionality reduction and CRC patients survival.
(DOCX)
