# Supplementary material for: 3’-UTR Polymorphisms in the MiRNA Machinery Genes DROSHA, DICER1, RAN, and XPO5 Are Associated with Colorectal Cancer Risk in a Korean Population
Source: PLoS One. 2015 Jul 6;10(7):e0131125. doi: 10.1371/journal.pone.0131125 (PMC4492935; doi:10.1371/journal.pone.0131125)
Supplement: S1 File — miRNA biogenesis gene genotype frequencies and CRC patient survival. Table B in S1 File. The combination of miRNA biogenesis genes polymorphisms based on MDR and CRC patients. Table C in S1 File. The combination of the polymorphisms of microRNA machinery genes in CRC patients and controls: male subgroup. Table D in S1 File. The combination of the polymorphisms of microRNA machinery genes in CRC patients and controls: female subgroup. Table E in S1 File. The combination of the polymorphisms of microRNA machinery genes between the subgroup for colorectal cancer and control subjects. Table F in S1 File. Genotype frequencies of miRNA biogenesis gene polymorphisms and CRC patients survival in TNM stage I + II. Table G in S1 File. Genotype frequencies of miRNA biogenesis genes polymorphisms and CRC patients survival in TNM stage III + IV. Table H in S1 File. Allele combinations of miRNA processing genes in CRC patients and controls using multifactor dimensionality reduction. Table I in S1 File. Allele combination of miRNA biogenesis genes polymorphisms based on multifactor dimensionality reduction and CRC patients survival. (DOCX) [file pone.0131125.s001.docx]

| **Table A in S1 File.** miRNA biogenesis gene genotype frequencies and CRC patient survival | | | | | | |  |  |  |
| --- | --- | --- | --- | --- | --- | --- | --- | --- | --- |
|  |  | Overall survival | | |  | Relapse-free survival***** | | | |
| Genotypes | Patients | Death | Adjusted HR^a^ | *P* | FDR^b^ | Relapse | Adjusted HR^a^ | *P* | FDR^b^ |
|  | (n=340) | (n=78) | (95% CI) |  |  | (n=70) | (95% CI) |  |  |
| ***DICER* rs3742330** |  |  |  |  |  |  |  |  |  |
| AA | 104 (30.6) | 20 (25.6) | 1.000 (reference) |  |  | 20 (28.6) | 1.000 (reference) |  |  |
| AG | 175 (51.5) | 42 (53.8) | 1.040 (0.586 - 1.846) | 0.893 | 0.893 | 36 (51.4) | 1.033 (0.562 - 1.901) | 0.917 | 0.917 |
| GG | 61 (17.9) | 16 (20.5) | 1.308 (0.623 - 2.744) | 0.48 | 0.553 | 14 (20.0) | 1.308 (0.590 - 2.901) | 0.51 | 0.765 |
| Dominant (AA vs. AG + GG) |  |  | 1.032 (0.603 - 1.767) | 0.909 | 0.909 |  | 1.025 (0.583 - 1.803) | 0.932 | 0.932 |
| Recessive (AA + AG vs. GG) |  |  | 1.200 (0.644 - 2.234) | 0.568 | 0.787 |  | 1.338 (0.690 - 2.594) | 0.391 | 0.587 |
|  |  |  |  |  |  |  |  |  |  |
| ***DROSHA* rs10719** |  |  |  |  |  |  |  |  |  |
| TT | 183 (53.8) | 37 (47.4) | 1.000 (reference) |  |  | 37 (52.9) | 1.000 (reference) |  |  |
| TC | 132 (38.8) | 37 (47.4) | 1.335 (0.804 - 2.218) | 0.266 | 0.667 | 27 (38.6) | 1.270 (0.734 - 2.199) | 0.396 | 0.917 |
| CC | 25 (7.4) | 4 (5.1) | 1.701 (0.554 - 5.228) | 0.356 | 0.553 | 6 (8.6) | 2.226 (0.787 - 6.300) | 0.134 | 0.402 |
| Dominant (TT vs. TC + CC) |  |  | 1.338 (0.815 - 2.195) | 0.252 | 0.768 |  | 1.347 (0.794 - 2.286) | 0.272 | 0.932 |
| Recessive (TT + TC vs. CC) |  |  | 1.154 (0.410 - 3.243) | 0.787 | 0.787 |  | 1.690 (0.655 - 4.357) | 0.28 | 0.587 |
|  |  |  |  |  |  |  |  |  |  |
| ***RAN* rs14035** |  |  |  |  |  |  |  |  |  |
| CC | 222 (65.3) | 50 (64.1) | 1.000 (reference) |  |  | 46 (65.7) | 1.000 (reference) |  |  |
| CT | 107 (31.5) | 27 (34.6) | 1.195 (0.714 - 1.999) | 0.5 | 0.667 | 21 (30.0) | 1.113 (0.623 - 1.989) | 0.72 | 0.917 |
| TT | 11 (3.2) | 1 (1.3) | 0.541 (0.072 - 4.070) | 0.553 | 0.553 | 3 (4.3) | 1.080 (0.236 - 4.935) | 0.922 | 0.922 |
| Dominant (CC vs. CT + TT) |  |  | 1.157 (0.696 - 1.922) | 0.576 | 0.768 |  | 1.077 (0.614 - 1.888) | 0.797 | 0.932 |
| Recessive (CC + CT vs. TT) |  |  | 0.518 (0.071 - 3.797) | 0.519 | 0.787 |  | 0.841 (0.189 - 3.754) | 0.822 | 0.822 |
|  |  |  |  |  |  |  |  |  |  |
| ***XPO5* rs11077** |  |  |  |  |  |  |  |  |  |
| AA | 277 (81.5) | 62 (79.5) | 1.000 (reference) |  |  | 54 (77.1) | 1.000 (reference) |  |  |
| AC | 62 (18.2) | 16 (20.5) | 1.249 (0.707 - 2.207) | 0.447 | 0.667 | 16 (22.9) | 1.198 (0.657 - 2.183) | 0.558 | 0.917 |
| CC | 1 (0.3) | 0 (0.0) | N/A | N/A | N/A | 0 (0.0) | N/A | N/A | N/A |
| Dominant (AA vs. AC + CC) |  |  | 1.235 (0.700 - 2.181) | 0.469 | 0.768 |  | 1.191 (0.654 - 2.170) | 0.569 | 0.932 |
| Recessive (AA + AC vs. CC) |  |  | N/A | N/A | N/A |  | N/A | N/A | N/A |

^*^Relapse-free survival that includes a group of patients who appear to have experienced a relapse. ^a^Adjusted for age, sex, tumor size, tumor site, tumor

differentiation, TNM stage, and chemotherapy.^b^False positive discovery rate (FDR)-adjusted *P*-value.

**Table B in S1 File.** The combination of miRNA biogenesis genes polymorphisms based on MDR and CRC patients

| Characteristics | Control (n= 400) | Case  (n=408) | COR(95% CI)^a^ | *P* | FDR^b^ | AOR(95% CI)^a^ | *P* | FDR^b^ |
| --- | --- | --- | --- | --- | --- | --- | --- | --- |
| ***DICER/DROSHA*** | | | | | | |  |  |
| AA/TT | 72 (18.0) | 66 (16.2) | 1.000 (reference) |  |  | 1.000 (reference) |  |  |
| AA/TC | 66 (16.5) | 53 (13.0) | 0.876 (0.536 - 1.433) | 0.598 | 0.973 | 0.826 (0.483 - 1.412) | 0.484 | 0.795 |
| AA/CC | 7 (1.8) | 6 (1.5) | 0.935 (0.299 - 2.925) | 0.908 | 0.973 | 1.024 (0.301 - 3.484) | 0.97 | 0.97 |
| AG/TT | 94 (23.5) | 116 (28.4) | 1.346 (0.875 - 2.071) | 0.176 | 0.704 | 1.272 (0.797 - 2.030) | 0.313 | 0.795 |
| AG/TC | 79 (19.8) | 73 (17.9) | 1.008 (0.636 - 1.599) | 0.973 | 0.973 | 0.883 (0.537 - 1.451) | 0.623 | 0.831 |
| AG/CC | 8 (2.0) | 18 (4.4) | 2.455 (1.001 - 6.021) | 0.05 | 0.4 | 2.359 (0.916 - 6.075) | 0.075 | 0.6 |
| GG/TT | 45 (11.3) | 42 (10.3) | 1.018 (0.595 - 1.742) | 0.948 | 0.973 | 0.912 (0.514 - 1.617) | 0.752 | 0.859 |
| GG/TC | 23 (5.8) | 28 (6.9) | 1.328 (0.697 - 2.531) | 0.389 | 0.973 | 1.272 (0.635 - 2.551) | 0.497 | 0.795 |
| GG/CC | 6 (1.5) | 6 (1.5) | 1.091 (0.335 - 3.550) | 0.885 | 0.973 | 0.593 (0.154 - 2.289) | 0.448 | 0.795 |
| ***DICER/RAN*** | | | | | | |  |  |
| AA/CC | 80 (20.0) | 76 (18.6) | 1.000 (reference) |  |  | 1.000 (reference) |  |  |
| AA/CT | 59 (14.8) | 46 (11.3) | 0.821 (0.499 - 1.349) | 0.436 | 0.698 | 0.671 (0.390 - 1.156) | 0.150 | 0.544 |
| AA/TT | 6 (1.5) | 3 (0.7) | 0.526 (0.127 - 2.180) | 0.376 | 0.698 | 0.329 (0.066 - 1.649) | 0.177 | 0.544 |
| AG/CC | 115 (28.8) | 138 (33.8) | 1.263 (0.847 - 1.884) | 0.252 | 0.672 | 1.165 (0.764 - 1.776) | 0.478 | 0.909 |
| AG/CT | 57 (14.3) | 60 (14.7) | 1.108 (0.686 - 1.790) | 0.675 | [0.771](file:///F:\1.%20CRC%20논문\논문교정한것\최종%20miRNA%20processing%20genes%20(CRC)%20최종1(table%20보충).xls#RANGE!A2) | 0.857 (0.504 - 1.456) | 0.568 | 0.909 |
| AG/TT | 9 (2.3) | 9 (2.2) | 1.053 (0.397 - 2.793) | 0.918 | 0.918 | 0.999 (0.335 - 2.978) | 0.999 | 0.999 |
| GG/CC | 38 (9.5) | 53 (13.0) | 1.468 (0.871 - 2.474) | 0.149 | 0.672 | 1.092 (0.619 - 1.926) | 0.762 | 0.921 |
| [GG/CT](file:///F:\1.%20CRC%20논문\논문교정한것\최종%20miRNA%20processing%20genes%20(CRC)%20최종1(table%20보충).xls#RANGE!A2) | 34 (8.5) | 22 (5.4) | [0.681 (0.366 - 1.268)](file:///F:\1.%20CRC%20논문\논문교정한것\최종%20miRNA%20processing%20genes%20(CRC)%20최종1(table%20보충).xls#RANGE!A2) | 0.226 | 0.672 | 0.646 (0.329 - 1.268) | 0.204 | 0.544 |
| GG/TT | 2 (0.5) | 1 (0.2) | 0.526 (0.047 - 5.925) | 0.603 | 0.771 | 0.723 (0.054 - 9.631) | 0.806 | 0.921 |
| ***DICER/XPO5*** | | | | | | |  |  |
| AA/AA | 123 (30.8) | 101 (24.8) | 1.000 (reference) |  |  | 1.000 (reference) |  |  |
| AA/AC | 22 (5.5) | 24 (5.9) | 1.329 (0.704 - 2.508) | 0.381 | 0.572 | 1.205 (0.610 - 2.380) | 0.592 | 0.775 |
| AA/CC | 0 (0.0) | 0 (0.0) | NA | NA | NA | NA | NA | NA |
| AG/AA | 151 (37.8) | 172 (42.2) | 1.387 (0.985 - 1.953) | 0.061 | 0.348 | 1.298 (0.903 - 1.867) | 0.159 | 0.670 |
| AG/AC | 29 (7.3) | 34 (8.3) | 1.428 (0.815 - 2.502) | 0.213 | 0.426 | 1.439 (0.798 - 2.595) | 0.227 | 0.670 |
| AG/CC | 1 (0.3) | 1 (0.2) | 1.218 (0.075 - 19.715) | 0.890 | 0.890 | 1.755 (0.096 - 32.148) | 0.704 | 0.775 |
| GG/AA | 63 (15.8) | 60 (14.7) | 1.160 (0.746 - 1.802) | 0.510 | 0.612 | 1.071 (0.670 - 1.711) | 0.775 | 0.775 |
| [GG/AC](file:///F:\1.%20CRC%20논문\논문교정한것\최종%20miRNA%20processing%20genes%20(CRC)%20최종1(table%20보충).xls#RANGE!A2) | 10 (2.5) | 16 (3.9) | [1.949 (0.847 - 4.481)](file:///F:\1.%20CRC%20논문\논문교정한것\최종%20miRNA%20processing%20genes%20(CRC)%20최종1(table%20보충).xls#RANGE!A2) | 0.116 | 0.348 | 1.560 (0.632 - 3.849) | 0.335 | 0.670 |
| GG/CC | 1 (0.3) | 0 (0.0) | NA | NA | NA | NA | NA | NA |
| ***DROSHA/RAN*** | | | | | | |  |  |
| TT/CC | 123 (30.8) | 139 (34.1) | 1.000 (reference) |  |  | 1.000 (reference) |  |  |
| TT/CT | 78 (19.5) | 78 (19.1) | 0.885 (0.595 - 1.316) | 0.546 | 0.637 | 0.840 (0.550 - 1.283) | 0.418 | 0.585 |
| TT/TT | 10 (2.5) | 7 (1.7) | 0.619 (0.229 - 1.677) | 0.346 | 0.484 | 0.716 (0.244 - 2.098) | 0.543 | 0.634 |
| TC/CC | 98 (24.5) | 105 (25.7) | 0.948 (0.657 - 1.368) | 0.776 | [0.776](file:///F:\1.%20CRC%20논문\논문교정한것\최종%20miRNA%20processing%20genes%20(CRC)%20최종1(table%20보충).xls#RANGE!A2) | 0.950 (0.646 - 1.399) | 0.796 | 0.796 |
| TC/CT | 63 (15.8) | 46 (11.3) | 0.646 (0.412 - 1.014) | 0.058 | 0.292 | 0.612 (0.375 - 1.000) | 0.050 | 0.282 |
| TC/TT | 7 (1.8) | 3 (0.7) | 0.379 (0.096 - 1.499) | 0.167 | 0.292 | 0.361 (0.077 - 1.688) | 0.195 | 0.341 |
| CC/CC | 12 (3.0) | 23 (5.6) | 1.696 (0.810 - 3.551) | 0.161 | 0.292 | 1.849 (0.850 - 4.023) | 0.121 | 0.282 |
| CC/CT | 9 (2.3) | 4 (1.0) | [0.393 (0.118 - 1.309)](file:///F:\1.%20CRC%20논문\논문교정한것\최종%20miRNA%20processing%20genes%20(CRC)%20최종1(table%20보충).xls#RANGE!A2) | 0.128 | 0.292 | 0.333 (0.090 - 1.229) | 0.099 | 0.282 |
| CC/TT | 0 (0.0) | 3 (0.7) | NA | NA | NA | NA | NA | NA |
| ***DROSHA/XPO5*** | | | | | | |  |  |
| TT/AA | 180 (45.0) | 185 (45.3) | 1.000 (reference) |  |  | 1.000 (reference) |  |  |
| TT/AC | 30 (7.5) | 38 (9.3) | 1.232 (0.732 - 2.075) | 0.432 | 0.867 | 1.229 (0.703 - 2.148) | 0.469 | 0.851 |
| TT/CC | 1 (0.3) | 1 (0.2) | 0.973 (0.060 - 15.675) | 0.985 | 0.985 | 1.419 (0.084 - 23.889) | 0.808 | 0.970 |
| TC/AA | 141 (35.3) | 128 (31.4) | 0.883 (0.645 - 1.211) | 0.440 | 0.867 | 0.852 (0.609 - 1.193) | 0.352 | 0.851 |
| TC/AC | 26 (6.5) | 26 (6.4) | 0.973 (0.544 - 1.740) | 0.926 | 0.985 | 0.993 (0.530 - 1.859) | 0.982 | 0.982 |
| TC/CC | 1 (0.3) | 0 (0.0) | NA | NA | NA | NA | NA | NA |
| CC/AA | 16 (4.0) | 20 (4.9) | 1.216 (0.611 - 2.422) | 0.578 | 0.867 | 1.332 (0.644 - 2.755) | 0.439 | 0.851 |
| [CC/AC](file:///F:\1.%20CRC%20논문\논문교정한것\최종%20miRNA%20processing%20genes%20(CRC)%20최종1(table%20보충).xls#RANGE!A2) | 5 (1.3) | 10 (2.5) | [1.946 (0.652 - 5.805)](file:///F:\1.%20CRC%20논문\논문교정한것\최종%20miRNA%20processing%20genes%20(CRC)%20최종1(table%20보충).xls#RANGE!A2) | 0.233 | 0.867 | 1.406 (0.438 - 4.518) | 0.567 | 0.851 |
| CC/CC | 0 (0.0) | 0 (0.0) | NA | NA | NA | NA | NA | NA |

^a^Adjusted odds ratio on the basis of risk factors, such as age, gender, hypertension, diabetes mellitus.

^b^False positive discovery rate (FDR)-adjusted *P*-value.

**Table C in S1 File.** The combination of the polymorphisms of microRNA machinery genes in CRC patients and controls: male subgroup

| Characteristics | Control | Colon | AOR(95% CI)^a^ | *P* | FDR^b^ | Rectum | AOR(95% CI) ^a^ | *P* | FDR^b^ |
| --- | --- | --- | --- | --- | --- | --- | --- | --- | --- |
|  | (n= 172) | (n=97) |  |  |  | (n=73) |  |  |  |
| ***DICER/DROSHA*** | | |  |  |  |  |  |  |  |
| AA/TT | 40 (23.3) | 16 (16.5) | 1.000 (reference) |  |  | 16 (21.9) | 1.000 (reference) |  |  |
| AA/TC | 25 (14.5) | 12 (12.4) | 1.275 (0.475 - 3.425) | 0.63 | 0.704 | 9 (12.3) | 0.920 (0.338 - 2.505) | 0.871 | 0.981 |
| AA/CC | 4 (2.3) | 2 (2.1) | 1.446 (0.215 - 9.733) | 0.704 | 0.704 | 1 (1.4) | 0.717 (0.056 - 9.216) | 0.798 | 0.981 |
| AG/TT | 41 (23.8) | 27 (27.8) | 1.962 (0.829 - 4.646) | 0.125 | 0.63 | 16 (21.9) | 0.990 (0.424 - 2.311) | 0.981 | 0.981 |
| AG/TC | 30 (17.4) | 18 (18.6) | 1.501 (0.629 - 3.581) | 0.36 | 0.63 | 10 (13.7) | 0.700 (0.260 - 1.884) | 0.48 | 0.981 |
| AG/CC | 2 (1.2) | 3 (3.1) | 3.430 (0.437 - 26.910) | 0.241 | 0.63 | 3 (4.1) | 3.215 (0.449 - 23.026) | 0.245 | 0.98 |
| GG/TT | 22 (12.8) | 15 (15.5) | 1.620 (0.617 - 4.252) | 0.328 | 0.63 | 8 (11.0) | 1.048 (0.371 - 2.963) | 0.93 | 0.981 |
| GG/TC | 6 (3.5) | 4 (4.1) | 1.342 (0.303 - 5.940) | 0.699 | 0.704 | 9 (12.3) | 3.301 (0.862 - 12.637) | 0.081 | 0.648 |
| GG/CC | 2 (1.2) | 0 (0.0) | NA | NA | NA | 1 (1.4) | 0.813 (0.057 - 11.636) | 0.879 | 0.981 |
| ***DICER/RAN*** | | | | | | |  |  |  |
| AA/CC | 36 (20.9) | 18 (18.6) | 1.000 (reference) |  |  | 16 (21.9) | 1.000 (reference) |  |  |
| AA/CT | 31 (18.0) | 10 (10.3) | 0.482 (0.176 - 1.323) | 0.157 | 0.716 | 10 (13.7) | 0.623 (0.232 - 1.675) | 0.348 | 0.745 |
| AA/TT | 2 (1.2) | 2 (2.1) | 0.854 (0.078 - 9.331) | 0.897 | 0.897 | 0 (0.0) | NA | NA | NA |
| AG/CC | 42 (24.4) | 37 (38.1) | 1.528 (0.692 - 3.371) | 0.294 | 0.716 | 20 (27.4) | 1.108 (0.476 - 2.578) | 0.813 | 0.848 |
| AG/CT | 28 (16.3) | 9 (9.3) | 0.518 (0.186 - 1.438) | 0.206 | 0.716 | 9 (12.3) | 0.360 (0.114 - 1.138) | 0.082 | 0.41 |
| AG/TT | 3 (1.7) | 2 (2.1) | 1.242 (0.170 - 9.080) | 0.831 | 0.897 | 0 (0.0) | NA | NA | NA |
| GG/CC | 16 (9.3) | 14 (14.4) | 1.114 (0.381 - 3.258) | 0.844 | 0.897 | 12 (16.4) | 1.520 (0.517 - 4.467) | 0.447 | 0.745 |
| GG/CT | 13 (7.6) | 4 (4.1) | 0.543 (0.148 - 1.999) | 0.358 | 0.716 | 6 (8.2) | 0.886 (0.257 - 3.050) | 0.848 | 0.848 |
| GG/TT | 1 (0.6) | 1 (1.0) | 1.854 (0.084 - 41.033) | 0.696 | 0.897 | 0 (0.0) | NA | NA | NA |
| ***DICER/XPO5*** | | | | | | |  |  |  |
| AA/AA | 62 (36.0) | 23 (23.7) | 1.000 (reference) |  |  | 19 (26.0) | 1.000 (reference) |  |  |
| AA/AC | 7 (4.1) | 7 (7.2) | 1.627 (0.454 - 5.827) | 0.454 | 0.693 | 7 (9.6) | 2.825 (0.818 - 9.751) | 0.1 | 0.25 |
| AA/CC | 0 (0.0) | 0 (0.0) | NA | NA | NA | 0 (0.0) | NA | NA | NA |
| AG/AA | 56 (32.6) | 38 (39.2) | 1.804 (0.920 - 3.537) | 0.086 | 0.215 | 24 (32.9) | 1.354 (0.650 - 2.823) | 0.418 | 0.697 |
| AG/AC | 16 (9.3) | 10 (10.3) | 1.354 (0.497 - 3.688) | 0.554 | 0.693 | 5 (6.8) | 1.002 (0.317 - 3.171) | 0.997 | 0.997 |
| AG/CC | 1 (0.6) | 0 (0.0) | NA | NA | NA | 0 (0.0) | NA | NA | NA |
| GG/AA | 24 (14.0) | 12 (12.4) | 0.987 (0.390 - 2.498) | 0.978 | 0.978 | 17 (23.3) | 2.158 (0.932 - 4.999) | 0.073 | 0.25 |
| GG/AC | 5 (2.9) | 7 (7.2) | 3.398 (0.930 - 12.415) | 0.064 | 0.215 | 1 (1.4) | 0.562 (0.059 - 5.370) | 0.616 | 0.77 |
| GG/CC | 1 (0.6) | 0 (0.0) | NA | NA | NA | 0 (0.0) | NA | NA | NA |
| ***DROSHA/XPO5*** | | | | | | |  |  |  |
| TT/AA | 84 (48.8) | 43 (44.3) | 1.000 (reference) |  |  | 34 (46.6) | 1.000 (reference) |  |  |
| TT/AC | 18 (10.5) | 15 (15.5) | 1.449 (0.606 - 3.466) | 0.404 | 0.848 | 6 (8.2) | 0.886 (0.318 - 2.469) | 0.817 | 0.936 |
| TT/CC | 1 (0.6) | 0 (0.0) | NA | NA | NA | 0 (0.0) | NA | NA | NA |
| TC/AA | 52 (30.2) | 27 (27.8) | 1.065 (0.562 - 2.017) | 0.848 | 0.848 | 23 (31.5) | 1.027 (0.533 - 1.980) | 0.936 | 0.936 |
| TC/AC | 8 (4.7) | 7 (7.2) | 1.511 (0.443 - 5.155) | 0.509 | 0.848 | 5 (6.8) | 1.446 (0.418 - 5.001) | 0.56 | 0.936 |
| TC/CC | 1 (0.6) | 0 (0.0) | NA | NA | NA | 0 (0.0) | NA | NA | NA |
| CC/AA | 6 (3.5) | 3 (3.1) | 1.563 (0.358 - 6.824) | 0.553 | 0.848 | 3 (4.1) | 1.110 (0.245 - 5.038) | 0.892 | 0.936 |
| CC/AC | 2 (1.2) | 2 (2.1) | 0.723 (0.079 - 6.587) | 0.773 | 0.848 | 2 (2.7) | 1.756 (0.220 - 14.026) | 0.596 | 0.936 |
| CC/CC | 0 (0.0) | 0 (0.0) | NA | NA | NA | 0 (0.0) | NA | NA | NA |

^a^Adjusted odds ratio on the basis of risk factors, such as age, gender, hypertension, diabetes mellitus.

^b^False positive discovery rate (FDR)-adjusted *P*-value.

**Table D in S1 File.** The combination of the polymorphisms of microRNA machinery genes in CRC patients and controls: female subgroup

| Characteristics | Control | Colon | OR(95% CI) | *P* | *FDR*^b^ | AOR(95% CI) ^a^ | *P* | *FDR*^b^ | Rectum | OR(95% CI) | *P* | *FDR*^b^ | AOR(95% CI) ^a^ | *P* | *FDR*^b^ |
| --- | --- | --- | --- | --- | --- | --- | --- | --- | --- | --- | --- | --- | --- | --- | --- |
|  | (n= 228) | (n=144) |  |  |  |  |  |  | (n=94) |  |  |  |  |  |  |
| ***DICER/DROSHA*** | | |  |  |  |  |  |  |  |  |  |  |  |  |  |
| AA/TT | 32 (14.0) | 19 (13.2) | 1.000 (reference) |  |  |  |  |  | 15 (16.0) | 1.000 (reference) |  |  |  |  |  |
| AA/TC | 41 (18.0) | 17 (11.8) | 0.698 (0.313 - 1.556) | 0.38 | 0.956 | 0.725 (0.299 - 1.759) | 0.478 | 0.879 | 15 (16.0) | 0.781 (0.333 - 1.830) | 0.569 | 0.759 | 0.521 (0.196 - 1.380) | 0.19 | 0.507 |
| AA/CC | 3 (1.3) | 1 (0.7) | 0.561 (0.054 - 5.789) | 0.628 | 0.956 | 0.464 (0.024 - 8.997) | 0.612 | 0.879 | 2 (2.1) | 1.422 (0.215 - 9.428) | 0.715 | 0.759 | 1.711 (0.167 - 17.496) | 0.651 | 0.868 |
| AG/TT | 53 (23.2) | 45 (31.3) | 1.430 (0.715 - 2.859) | 0.312 | 0.956 | 1.509 (0.700 - 3.252) | 0.294 | 0.879 | 28 (29.8) | 1.127 (0.524 - 2.423) | 0.759 | 0.759 | 0.915 (0.401 - 2.088) | 0.832 | 0.951 |
| AG/TC | 49 (21.5) | 30 (20.8) | 1.031 (0.498 - 2.134) | 0.934 | 0.956 | 0.910 (0.413 - 2.004) | 0.814 | 0.879 | 15 (16.0) | 0.653 (0.281 - 1.517) | 0.322 | 0.759 | 0.447 (0.173 - 1.151) | 0.095 | 0.396 |
| AG/CC | 6 (2.6) | 7 (4.9) | 1.965 (0.575 - 6.718) | 0.282 | 0.956 | 1.989 (0.495 - 7.991) | 0.333 | 0.879 | 5 (5.3) | 1.778 (0.467 - 6.762) | 0.399 | 0.759 | 1.807 (0.421 - 7.754) | 0.426 | 0.682 |
| GG/TT | 23 (10.1) | 14 (9.7) | 1.025 (0.428 - 2.457) | 0.956 | 0.956 | 0.810 (0.293 - 2.242) | 0.686 | 0.879 | 5 (5.3) | 0.464 (0.148 - 1.458) | 0.189 | 0.759 | 0.339 (0.094 - 1.224) | 0.099 | 0.396 |
| GG/TC | 17 (7.5) | 9 (6.3) | 0.892 (0.332 - 2.394) | 0.82 | 0.956 | 1.126 (0.367 - 3.450) | 0.836 | 0.879 | 6 (6.4) | 0.753 (0.247 - 2.295) | 0.618 | 0.759 | 0.545 (0.138 - 2.143) | 0.385 | 0.682 |
| GG/CC | 4 (1.8) | 2 (1.4) | 0.842 (0.141 - 5.043) | 0.851 | 0.956 | 0.835 (0.082 - 8.473) | 0.879 | 0.879 | 3 (3.2) | 1.600 (0.317 - 8.067) | 0.569 | 0.759 | 0.939 (0.101 - 8.730) | 0.956 | 0.956 |
| ***DICER/RAN*** | | | | | | |  |  |  |  |  |  |  |  |  |
| AA/CC | 44 (19.3) | 19 (13.2) | 1.000 (reference) |  |  |  |  |  | 23 (24.5) | 1.000 (reference) |  |  |  |  |  |
| AA/CT | 28 (12.3) | 18 (12.5) | 1.489 (0.669 - 3.314) | 0.33 | 0.396 | 1.179 (0.477 - 2.913) | 0.721 | 0.855 | 8 (8.5) | 0.547 (0.215 - 1.390) | 0.205 | 0.707 | 0.404 (0.138 - 1.183) | 0.098 | 0.343 |
| AA/TT | 4 (1.8) | 0 (0.0) | NA | NA | NA | NA | NA | NA | 1 (1.1) | 0.478 (0.051 - 4.531) | 0.52 | 0.749 | 0.537 (0.053 - 5.467) | 0.599 | 0.699 |
| AG/CC | 73 (32.0) | 50 (34.7) | 1.586 (0.830 - 3.030) | 0.162 | 0.335 | 1.415 (0.724 - 2.766) | 0.31 | 0.59 | 31 (33.0) | 0.812 (0.421 - 1.566) | 0.535 | 0.749 | 0.705 (0.352 - 1.412) | 0.324 | 0.49 |
| AG/CT | 29 (12.7) | 26 (18.1) | 2.076 (0.976 - 4.417) | 0.058 | 0.335 | 1.776 (0.776 - 4.065) | 0.174 | 0.522 | 16 (17.0) | 1.056 (0.478 - 2.330) | 0.894 | 0.921 | 0.841 (0.348 - 2.031) | 0.7 | 0.7 |
| AG/TT | 6 (2.6) | 6 (4.2) | 2.316 (0.662 - 8.107) | 0.189 | 0.335 | 2.971 (0.728- 12.122) | 0.129 | 0.522 | 1 (1.1) | 0.319 (0.036 - 2.810) | 0.303 | 0.707 | 0.247 (0.023 - 2.614) | 0.245 | 0.49 |
| GG/CC | 22 (9.6) | 16 (11.1) | 1.684 (0.728 - 3.898) | 0.223 | 0.335 | 1.504 (0.590 - 3.835) | 0.393 | 0.59 | 11 (11.7) | 0.957 (0.396 - 2.311) | 0.921 | 0.921 | 0.615 (0.222 - 1.703) | 0.35 | 0.49 |
| GG/CT | 21 (9.2) | 9 (6.3) | 0.993 (0.385 - 2.562) | 0.988 | 0.396 | 0.901 (0.295 - 2.751) | 0.855 | 0.855 | 3 (3.2) | 0.273 (0.074 - 1.014) | 0.052 | 0.364 | 0.316 (0.081 - 1.236) | 0.098 | 0.343 |
| GG/TT | 1 (0.4) | 0 (0.0) | NA | NA | NA | NA | NA | NA | 0 (0.0) | NA | NA | NA | NA | NA | NA |
| ***DICER/XPO5*** | | | | | | |  |  |  |  |  |  |  |  |  |
| AA/AA | 61 (26.8) | 31 (21.5) | 1.000 (reference) |  |  |  |  |  | 28 (29.8) | 1.000 (reference) |  |  |  |  |  |
| AA/AC | 15 (6.6) | 6 (4.2) | 0.787 (0.278 - 2.229) | 0.652 | 0.828 | 0.799 (0.248 - 2.578) | 0.708 | 0.851 | 4 (4.3) | 0.581 (0.177 - 1.910) | 0.371 | 0.618 | 0.511 (0.139 - 1.875) | 0.312 | 0.78 |
| AA/CC | 0 (0.0) | 0 (0.0) | NA | NA | NA | NA | NA | NA | 0 (0.0) | NA | NA | NA | NA | NA | NA |
| AG/AA | 95 (41.7) | 69 (47.9) | 1.429 (0.840 - 2.433) | 0.188 | 0.48 | 1.375 (0.782 - 2.416) | 0.268 | 0.67 | 41 (43.6) | 0.940 (0.527 - 1.676) | 0.835 | 0.835 | 0.853 (0.463 - 1.572) | 0.61 | 0.896 |
| AG/AC | 13 (5.7) | 12 (8.3) | 1.816 (0.742 - 4.449) | 0.192 | 0.48 | 1.974 (0.753 - 5.179) | 0.167 | 0.67 | 7 (7.4) | 1.173 (0.422 - 3.260) | 0.76 | 0.835 | 1.043 (0.346 - 3.147) | 0.941 | 0.941 |
| AG/CC | 0 (0.0) | 1 (0.7) | 0.000 (0.000 - 0.000) | NA | NA | NA | NA | NA | 0 (0.0) | NA | NA | NA | NA | NA | 0.62 |
| GG/AA | 39 (17.1) | 22 (15.3) | 1.110 (0.563 - 2.187) | 0.763 | 0.828 | 1.094 (0.518 - 2.310) | 0.813 | 0.851 | 9 (9.6) | 0.503 (0.215 - 1.178) | 0.114 | 0.57 | 0.495 (0.202 - 1.213) | 0.124 | NA |
| GG/AC | 5 (2.2) | 3 (2.1) | 1.181 (0.265 - 5.266) | 0.828 | 0.828 | 0.842 (0.141 - 5.048) | 0.851 | 0.851 | 5 (5.3) | 2.179 (0.583 - 8.138) | 0.247 | 0.618 | 1.345 (0.271 - 6.664) | 0.717 | 0.896 |
| GG/CC | 0 (0.0) | 0 (0.0) | NA | NA | NA | NA | NA | NA | 0 (0.0) | NA | NA | NA | NA | NA | NA |
| ***DROSHA/RAN*** | | | | | | |  |  |  |  |  |  |  |  |  |
| TT/CC | 66 (28.9) | 42 (29.2) | 1.000 (reference) |  |  |  |  |  | 33 (35.1) | 1.000 (reference) |  |  |  |  |  |
| TT/CT | 37 (16.2) | 32 (22.2) | 1.359 (0.738 - 2.504) | 0.325 | 0.934 | 1.193 (0.612 - 2.326) | 0.604 | 0.835 | 14 (14.9) | 0.757 (0.360 - 1.592) | 0.463 | 0.463 | 0.838 (0.384 - 1.833) | 0.659 | 0.659 |
| TT/TT | 5 (2.2) | 4 (2.8) | 1.257 (0.319 - 4.950) | 0.744 | 0.934 | 1.964 (0.451 - 8.542) | 0.368 | 0.835 | 1 (1.1) | 0.400 (0.045 - 3.565) | 0.412 | 0.463 | 0.592 (0.063 - 5.599) | 0.648 | 0.659 |
| TC/CC | 64 (28.1) | 37 (25.7) | 0.909 (0.519 - 1.590) | 0.737 | 0.934 | 0.894 (0.499 - 1.599) | 0.705 | 0.835 | 22 (23.4) | 0.688 (0.363 - 1.304) | 0.251 | 0.463 | 0.648 (0.333 - 1.261) | 0.202 | 0.52 |
| TC/CT | 37 (16.2) | 18 (12.5) | 0.765 (0.386 - 1.514) | 0.441 | 0.934 | 0.758 (0.372 - 1.544) | 0.445 | 0.835 | 13 (13.8) | 0.703 (0.329 - 1.499) | 0.361 | 0.463 | 0.620 (0.270 - 1.424) | 0.26 | 0.52 |
| TC/TT | 6 (2.6) | 1 (0.7) | 0.262 (0.030 - 2.253) | 0.222 | 0.934 | 0.302 (0.033 - 2.724) | 0.286 | 0.835 | 1 (1.1) | 0.333 (0.039 - 2.884) | 0.318 | 0.463 | 0.349 (0.038 - 3.207) | 0.352 | 0.528 |
| CC/CC | 9 (3.9) | 6 (4.2) | 1.048 (0.348 - 3.157) | 0.934 | 0.934 | 0.994 (0.307 - 3.219) | 0.992 | 0.992 | 10 (10.6) | 2.222 (0.823 - 5.997) | 0.115 | 0.463 | 2.283 (0.803 - 6.488) | 0.121 | 0.52 |
| CC/CT | 4 (1.8) | 3 (2.1) | 1.179 (0.251 - 5.532) | 0.835 | 0.934 | 1.361 (0.258 - 7.176) | 0.716 | 0.835 | 0 (0.0) | NA | NA | NA | NA | NA | NA |
| CC/TT | 0 (0.0) | 1 (0.7) | NA | NA | NA | NA | NA | NA | 0 (0.0) | NA | NA | NA | NA | NA | NA |
| ***DROSHA/XPO5*** | | | | | | |  |  |  |  |  |  |  |  |  |
| TT/AA | 96 (42.1) | 67 (46.5) | 1.000 (reference) |  |  |  |  |  | 41 (43.6) | 1.000 (reference) |  |  |  |  |  |
| TT/AC | 12 (5.3) | 10 (6.9) | 1.194 (0.488 - 2.923) | 0.698 | 0.873 | 1.069 (0.391 - 2.924) | 0.896 | 0.896 | 7 (7.4) | 1.366 (0.502 - 3.718) | 0.542 | 0.625 | 1.243 (0.426 - 3.626) | 0.69 | 0.69 |
| TT/CC | 0 (0.0) | 1 (0.7) | NA | NA | NA | NA | NA |  | 0 (0.0) | 0.000 (0.000 - 0.000) | NA | NA | NA | NA | NA |
| TC/AA | 89 (39.0) | 48 (33.3) | 0.773 (0.483 - 1.236) | 0.282 | 0.8 | 0.737 (0.446 - 1.217) | 0.232 | 0.896 | 30 (31.9) | 0.789 (0.454 - 1.371) | 0.401 | 0.625 | 0.663 (0.366 - 1.200) | 0.175 | 0.528 |
| TC/AC | 18 (7.9) | 8 (5.6) | 0.637 (0.262 - 1.550) | 0.32 | 0.8 | 0.766 (0.290 - 2.024) | 0.59 | 0.896 | 6 (6.4) | 0.781 (0.289 - 2.108) | 0.625 | 0.625 | 0.601 (0.200 - 1.808) | 0.365 | 0.528 |
| TC/CC | 0 (0.0) | 0 (0.0) | NA | NA | NA | NA | NA |  | 0 (0.0) | 0.000 (0.000 - 0.000) | NA | NA | NA | NA | NA |
| CC/AA | 10 (4.4) | 7 (4.9) | 1.003 (0.363 - 2.768) | 0.996 | 0.996 | 1.113 (0.365 - 3.393) | 0.85 | 0.896 | 7 (7.4) | 1.639 (0.584 - 4.604) | 0.348 | 0.625 | 1.713 (0.570 - 5.147) | 0.338 | 0.528 |
| CC/AC | 3 (1.3) | 3 (2.1) | 1.433 (0.281 - 7.316) | 0.666 | 0.873 | 1.233 (0.174 - 8.726) | 0.834 | 0.896 | 3 (3.2) | 2.342 (0.454 - 12.09) | 0.31 | 0.625 | 2.109 (0.341 - 13.052) | 0.422 | 0.528 |
| CC/CC | 0 (0.0) | 0 (0.0) | NA | NA | NA | NA | NA | NA | 0 (0.0) | 0.000 (0.000 - 0.000) | NA | NA | NA | NA | NA |
| ***RAN/XPO5*** | | | | | | |  |  |  |  |  |  |  |  |  |
| CC/AA | 117 (51.3) | 75 (52.1) | 1.000 (reference) |  |  |  |  |  | 52 (55.3) | 1.000 (reference) |  |  |  |  |  |
| CC/AC | 22 (9.6) | 9 (6.3) | 0.638 (0.279 - 1.461) | 0.288 | 0.72 | 0.647 (0.274 - 1.529) | 0.321 | 0.803 | 13 (13.8) | 1.330 (0.622 - 2.842) | 0.462 | 0.616 | 1.215 (0.550 - 2.684) | 0.631 | 0.631 |
| CC/CC | 0 (0.0) | 1 (0.7) | NA | NA | NA | NA | NA | NA | 0 (0.0) | NA | NA | NA | NA | NA | NA |
| CT/AA | 69 (30.3) | 42 (29.2) | 0.950 (0.587 - 1.536) | 0.833 | 0.84 | 0.914 (0.549 - 1.524) | 0.731 | 0.972 | 24 (25.5) | 0.783 (0.444 - 1.381) | 0.398 | 0.616 | 0.791 (0.437 - 1.431) | 0.438 | 0.631 |
| CT/AC | 9 (3.9) | 11 (7.6) | 1.907 (0.754 - 4.820) | 0.173 | 0.72 | 1.833 (0.687 - 4.889) | 0.226 | 0.803 | 3 (3.2) | 0.750 (0.195 - 2.884) | 0.676 | 0.676 | 0.695 (0.173 - 2.787) | 0.608 | 0.631 |
| CT/CC | 0 (0.0) | 0 (0.0) | NA | NA | NA | NA | NA | NA | 0 (0.0) | NA | NA | NA | NA | NA | NA |
| TT/AA | 9 (3.9) | 5 (3.5) | 0.867 (0.280 - 2.686) | 0.804 | 0.84 | 0.979 (0.305 - 3.147) | 0.972 | 0.972 | 2 (2.1) | 0.500 (0.104 - 2.395) | 0.386 | 0.616 | 0.494 (0.100 - 2.437) | 0.386 | 0.631 |
| TT/AC | 2 (0.9) | 1 (0.7) | 0.780 (0.070 - 8.753) | 0.84 | 0.84 | 1.151 (0.094- 14.091) | 0.912 | 0.972 | 0 (0.0) | NA | NA | NA | NA | NA | NA |
| TT/CC | 0 (0.0) | 0 (0.0) | NA | NA | NA | NA | NA | NA | 0 (0.0) | NA | NA | NA | NA | NA | NA |

^a^Adjusted odds ratio on the basis of risk factors, such as age, gender, hypertension, diabetes mellitus.

^b^False positive discovery rate (FDR)-adjusted *P*-value.

**Table E in S1 File.** The combination of the polymorphisms of microRNA machinery genes between the subgroup for colorectal cancer and control subjects

| Characteristics | Control | Colon | COR(95% CI) | *P* | *FDR*^b^ | AOR(95% CI)^a^ | *P* | *FDR*^b^ | Rectum | COR(95% CI) | *P* | *FDR*^b^ | AOR(95% CI)^a^ | *P* | *FDR*^b^ |
| --- | --- | --- | --- | --- | --- | --- | --- | --- | --- | --- | --- | --- | --- | --- | --- |
|  | (n= 400) | (n=241) |  |  |  |  |  |  | (n=167) |  |  |  |  |  |  |
| ***DICER/DROSHA*** | | |  |  |  |  |  |  |  |  |  |  |  |  |  |
| AA/TT | 72 (18.0) | 35 (14.5) | 1.000 (reference) |  |  |  |  |  | 31 (18.6) | 1.000 (reference) |  |  |  |  |  |
| AA/TC | 66 (16.5) | 29 (12.0) | 0.904 (0.499 - 1.639) | 0.739 | 0.845 | 0.934 (0.487 - 1.793) | 0.838 | 0.958 | 24 (14.4) | 0.845 (0.450 - 1.584) | 0.599 | 0.799 | 0.728 (0.368 - 1.439) | 0.361 | 0.722 |
| AA/CC | 7 (1.8) | 3 (1.2) | 0.882 (0.215 - 3.617) | 0.861 | 0.861 | 1.023 (0.221 - 4.742) | 0.977 | 0.977 | 3 (1.8) | 0.996 (0.242 - 4.104) | 0.995 | 0.995 | 1.052 (0.232 - 4.780) | 0.947 | 0.955 |
| AG/TT | 94 (23.5) | 72 (29.9) | 1.576 (0.949 - 2.617) | 0.079 | 0.316 | 1.649 (0.937 - 2.901) | 0.083 | 0.492 | 44 (26.3) | 1.087 (0.626 - 1.889) | 0.767 | 0.877 | 0.983 (0.547 - 1.767) | 0.955 | 0.955 |
| AG/TC | 79 (19.8) | 48 (19.9) | 1.250 (0.728 - 2.145) | 0.418 | 0.836 | 1.152 (0.644 - 2.061) | 0.635 | 0.958 | 25 (15.0) | 0.735 (0.397 - 1.361) | 0.327 | 0.654 | 0.605 (0.311 - 1.177) | 0.139 | 0.664 |
| AG/CC | 8 (2.0) | 10 (4.1) | 2.571 (0.933 - 7.086) | 0.068 | 0.316 | 2.392 (0.789 - 7.251) | 0.123 | 0.492 | 8 (4.8) | 2.323 (0.799 - 6.748) | 0.122 | 0.654 | 2.019 (0.660 - 6.179) | 0.218 | 0.664 |
| GG/TT | 45 (11.3) | 29 (12.0) | 1.326 (0.715 - 2.458) | 0.371 | 0.836 | 1.131 (0.569 - 2.247) | 0.726 | 0.958 | 13 (7.8) | 0.671 (0.318 - 1.416) | 0.295 | 0.654 | 0.637 (0.296 - 1.372) | 0.249 | 0.664 |
| GG/TC | 23 (5.8) | 13 (5.4) | 1.163 (0.527 - 2.565) | 0.709 | 0.845 | 1.176 (0.489 - 2.832) | 0.717 | 0.958 | 15 (9.0) | 1.515 (0.698 - 3.287) | 0.294 | 0.654 | 1.264 (0.547 - 2.919) | 0.583 | 0.933 |
| GG/CC | 6 (1.5) | 2 (0.8) | 0.686 (0.132 - 3.572) | 0.654 | 0.845 | 0.490 (0.074 - 3.246) | 0.46 | 0.958 | 4 (2.4) | 1.548 (0.408 - 5.874) | 0.52 | 0.799 | 0.914 (0.205 - 4.081) | 0.906 | 0.955 |
| ***DICER/RAN*** | | | | | | |  |  |  |  |  |  |  |  |  |
| AA/CC | 80 (20.0) | 39 (23.4) | 1.000 (reference) | 0 | 0 | 0 | 0 | 0 | 39 (23.4) | 1.000 (reference) |  |  |  |  |  |
| AA/CT | 59 (14.8) | 18 (10.8) | 0.626 (0.326 - 1.201) | 0.159 | 0.392 | 0.532 (0.262 - 1.082) | 0.081 | 0.345 | 18 (10.8) | 0.626 (0.326 - 1.201) | 0.159 | 0.392 | 0.532 (0.262 - 1.082) | 0.081 | 0.345 |
| AA/TT | 6 (1.5) | 1 (0.6) | 0.342 (0.040 - 2.939) | 0.328 | 0.574 | 0.301 (0.032 - 2.803) | 0.291 | 0.407 | 1 (0.6) | 0.342 (0.040 - 2.939) | 0.328 | 0.574 | 0.301 (0.032 - 2.803) | 0.291 | 0.407 |
| AG/CC | 115 (28.8) | 51 (30.5) | 0.910 (0.549 - 1.508) | 0.714 | 0.733 | 0.836 (0.491 - 1.424) | 0.51 | 0.595 | 51 (30.5) | 0.910 (0.549 - 1.508) | 0.714 | 0.733 | 0.836 (0.491 - 1.424) | 0.51 | 0.595 |
| AG/CT | 57 (14.3) | 25 (15.0) | 0.900 (0.491 - 1.650) | 0.733 | 0.733 | 0.636 (0.320 - 1.264) | 0.197 | 0.345 | 25 (15.0) | 0.900 (0.491 - 1.650) | 0.733 | 0.733 | 0.636 (0.320 - 1.264) | 0.197 | 0.345 |
| AG/TT | 9 (2.3) | 1 (0.6) | 0.228 (0.028 - 1.863) | 0.168 | 0.392 | 0.159 (0.017 - 1.514) | 0.11 | 0.345 | 1 (0.6) | 0.228 (0.028 - 1.863) | 0.168 | 0.392 | 0.159 (0.017 - 1.514) | 0.11 | 0.345 |
| GG/CC | 38 (9.5) | 23 (13.8) | 1.242 (0.652 - 2.364) | 0.51 | 0.714 | 0.858 (0.420 - 1.753) | 0.674 | 0.674 | 23 (13.8) | 1.242 (0.652 - 2.364) | 0.51 | 0.714 | 0.858 (0.420 - 1.753) | 0.674 | 0.674 |
| GG/CT | 34 (8.5) | 9 (5.4) | 0.534 (0.237 - 1.243) | 0.149 | 0.392 | 0.549 (0.228 - 1.326) | 0.183 | 0.345 | 9 (5.4) | 0.543 (0.237 - 1.243) | 0.149 | 0.392 | 0.549 (0.228 - 1.326) | 0.183 | 0.345 |
| GG/TT | 2 (0.5) | 0 (0.0) | NA | NA | NA | NA | NA | 0 | 0 (0.0) | NA | NA | NA | NA | NA |  |
| ***DICER/XPO5*** | | | | | | | 0 | 0 |  |  |  |  |  |  |  |
| AA/AA | 123 (30.8) | 47 (28.1) | 1.000 (reference) | 0 | 0 | 0 | 0 | 0 | 47 (28.1) | 1.000 (reference) |  |  |  |  |  |
| AA/AC | 22 (5.5) | 11 (6.6) | 1.309 (0.589 - 2.906) | 0.509 | 0.836 | 1.271 (0.547 - 2.950) | 0.577 | 0.963 | 11 (6.6) | 1.309 (0.589 - 2.906) | 0.509 | 0.836 | 1.271 (0.547 - 2.950) | 0.577 | 0.963 |
| AA/CC | 0 (0.0) | 0 (0.0) | NA | NA | NA | NA | NA | NA | 0 (0.0) | NA | NA | NA | NA | NA | NA |
| AG/AA | 151 (37.8) | 65 (38.9) | 1.127 (0.722 - 1.757) | 0.599 | 0.836 | 1.034 (0.648 - 1.652) | 0.888 | 0.963 | 65 (38.9) | 1.127 (0.722 - 1.757) | 0.599 | 0.836 | 1.034 (0.648 - 1.652) | 0.888 | 0.963 |
| AG/AC | 29 (7.3) | 12 (7.2) | 1.083 (0.511 - 2.297) | 0.836 | 0.836 | 1.031 (0.468 - 2.272) | 0.941 | 0.963 | 12 (7.2) | 1.083 (0.511 - 2.297) | 0.836 | 0.836 | 1.031 (0.468 - 2.272) | 0.941 | 0.963 |
| AG/CC | 1 (0.3) | 0 (0.0) | NA | NA | NA | NA | NA | NA | 0 (0.0) | NA | NA | NA | NA | NA | NA |
| GG/AA | 63 (15.8) | 26 (15.6) | 1.080 (0.613 - 1.904) | 0.79 | 0.836 | 1.018 (0.563 - 1.841) | 0.953 | 0.963 | 26 (15.6) | 1.080 (0.613 - 1.904) | 0.79 | 0.836 | 1.018 (0.563 - 1.841) | 0.953 | 0.963 |
| GG/AC | 10 (2.5) | 6 (3.6) | 1.570 (0.541 -4.562) | 0.407 | 0.836 | 1.029 (0.308 - 3.436) | 0.963 | 0.963 | 6 (3.6) | 1.570 (0.541 - 4.562) | 0.407 | 0.836 | 1.029 (0.308 - 3.436) | 0.963 | 0.963 |
| GG/CC | 1 (0.3) | 0 (0.0) | NA | NA | NA | NA | NA | NA | 0 (0.0) | NA | NA | NA | NA | NA | NA |
| ***DROSHA/RAN*** | | | | | | | 0 | 0 |  |  |  |  |  |  |  |
| TT/CC | 123 (30.8) | 56 (33.5) | 1.000 (reference) | 0 | 0 | 0 | 0 | 0 | 56 (33.5) | 1.000 (reference) |  |  |  |  |  |
| TT/CT | 78 (19.5) | 31 (18.6) | 0.873 (0.518 - 1.472) | 0.61 | 0.712 | 0.870 (0.506 - 1.497) | 0.615 | 0.718 | 31 (18.6) | 0.873 (0.518 - 1.472) | 0.61 | 0.712 | 0.870 (0.506 - 1.497) | 0.615 | 0.718 |
| TT/TT | 10 (2.5) | 1 (0.6) | 0.220 (0.027 - 1.758) | 0.153 | 0.398 | 0.290 (0.035 - 2.403) | 0.251 | 0.438 | 1 (0.6) | 0.220 (0.027 - 1.758) | 0.153 | 0.398 | 0.290 (0.035 - 2.403) | 0.251 | 0.438 |
| TC/CC | 98 (24.5) | 43 (25.7) | 0.964 (0.598 - 1.554) | 0.88 | 0.88 | 0.921 (0.559 - 1.515) | 0.745 | 0.745 | 43 (25.7) | 0.964 (0.598 - 1.554) | 0.88 | 0.88 | 0.921 (0.559 - 1.515) | 0.745 | 0.745 |
| TC/CT | 63 (15.8) | 20 (12.0) | 0.697 (0.385 - 1.263) | 0.234 | 0.398 | 0.620 (0.326 - 1.179) | 0.145 | 0.438 | 20 (12.0) | 0.697 (0.385 - 1.263) | 0.234 | 0.398 | 0.620 (0.326 - 1.179) | 0.145 | 0.438 |
| TC/TT | 7 (1.8) | 1 (0.6) | 0.314 (0.038 - 2.612) | 0.284 | 0.398 | 0.320 (0.035 - 2.919) | 0.313 | 0.438 | 1 (0.6) | 0.314 (0.038 - 2.612) | 0.284 | 0.398 | 0.320 (0.035 - 2.919) | 0.313 | 0.438 |
| CC/CC | 12 (3.0) | 14 (8.4) | 2.563 (1.114 - 5.896) | 0.027 | 0.189 | 2.419 (1.000 - 5.848) | 0.05 | 0.35 | 14 (8.4) | 2.563 (1.114 - 5.896) | 0.027 | 0.189 | 2.419 (1.000 - 5.848) | 0.05 | 0.35 |
| CC/CT | 9 (2.3) | 1 (0.6) | 0.244 (0.030 - 1.973) | 0.186 | 0.398 | 0.241 (0.028 - 2.051) | 0.193 | 0.438 | 1 (0.6) | 0.244 (0.030 - 1.973) | 0.186 | 0.398 | 0.241 (0.028 - 2.051) | 0.193 | 0.438 |
| CC/TT | 0 (0.0) | 0 (0.0) | NA | NA | NA | NA | NA | NA | 0 (0.0) | NA | NA | NA | NA | NA | NA |
| ***DROSHA/XPO5*** | | | | | | | 0 | 0 |  |  |  |  |  |  |  |
| TT/AA | 180 (45.0) | 75 (44.9) | 1.000 (reference) | 0 | 0 | 0 | 0 | 0 | 75 (44.9) | 1.000 (reference) |  |  |  |  |  |
| TT/AC | 30 (7.5) | 13 (7.8) | 1.040 (0.514 - 2.104) | 0.913 | 0.968 | 1.046 (0.504 - 2.171) | 0.905 | 0.905 | 13 (7.8) | 1.040 (0.514 - 2.104) | 0.913 | 0.968 | 1.046 (0.504 - 2.171) | 0.905 | 0.905 |
| TT/CC | 1 (0.3) | 0 (0.0) | NA | NA | NA | NA | NA | NA | 0 (0.0) | NA | NA | NA | NA | NA | NA |
| TC/AA | 141 (35.3) | 53 (31.7) | 0.902 (0.596 - 1.366) | 0.627 | 0.968 | 0.829 (0.536 - 1.283) | 0.4 | 0.667 | 53 (31.7) | 0.902 (0.596 - 1.366) | 0.627 | 0.968 | 0.829 (0.536 - 1.283) | 0.4 | 0.667 |
| TC/AC | 26 (6.5) | 11 (6.6) | 1.015 (0.477 - 2.160) | 0.968 | 0.968 | 0.924 (0.415 - 2.058) | 0.846 | 0.905 | 11 (6.6) | 1.015 (0.477 - 2.160) | 0.968 | 0.968 | 0.924 (0.415 - 2.058) | 0.846 | 0.905 |
| TC/CC | 1 (0.3) | 0 (0.0) | NA | NA | NA | NA | NA | NA | 0 (0.0) | NA | NA | NA | NA | NA | NA |
| CC/AA | 16 (4.0) | 10 (6.0) | 1.500 (0.651 - 3.456) | 0.341 | 0.853 | 1.466 (0.614 - 3.500) | 0.389 | 0.667 | 10 (6.0) | 1.500 (0.651 - 3.456) | 0.341 | 0.853 | 1.466 (0.614 - 3.500) | 0.389 | 0.667 |
| CC/AC | 5 (1.3) | 5 (3.0) | 2.400 (0.675 -8.534) | 0.176 | 0.853 | 1.862 (0.486 - 7.126) | 0.364 | 0.667 | 5 (3.0) | 2.400 (0.675 - 8.534) | 0.176 | 0.853 | 1.862 (0.486 - 7.126) | 0.364 | 0.667 |
| CC/CC | 0 (0.0) | 0 (0.0) | NA | NA | NA | NA | NA | NA | 0 (0.0) | NA | NA | NA | NA | NA | NA |
| ***RAN/XPO5*** | | | | | | | 0 | 0 |  |  |  |  |  |  |  |
| CC/AA | 191 (47.8) | 92 (55.1) | 1.000 (reference) | 0 | 0 | 0 | 0 | 0 | 92 (55.1) | 1.000 (reference) |  |  |  |  |  |
| CC/AC | 41 (10.3) | 21 (12.6) | 1.063 (0.594 - 1.903) | 0.836 | 0.959 | 0.985 (0.537 - 1.809) | 0.962 | 0.962 | 21 (12.6) | 1.063 (0.594 - 1.903) | 0.836 | 0.959 | 0.985 (0.537 - 1.809) | 0.962 | 0.962 |
| CC/CC | 1 (0.3) | 0 (0.0) | NA | NA | NA | NA | NA | NA | 0 (0.0) | NA | NA | NA | NA | NA | NA |
| CT/AA | 132 (33.0) | 44 (26.3) | 0.692 (0.454 - 1.056) | 0.088 | 0.226 | 0.650 (0.418 - 1.011) | 0.056 | 0.224 | 44 (26.3) | 0.692 (0.454 - 1.056) | 0.088 | 0.226 | 0.650 (0.418 - 1.011) | 0.056 | 0.224 |
| CT/AC | 17 (4.3) | 8 (4.8) | 0.977 (0.407 - 2.347) | 0.959 | 0.959 | 0.863 (0.347 - 2.147) | 0.752 | 0.962 | 8 (4.8) | 0.977 (0.407 - 2.347) | 0.959 | 0.959 | 0.863 (0.347 - 2.147) | 0.752 | 0.962 |
| CT/CC | 1 (0.3) | 0 (0.0) | NA | NA | NA | NA | NA | NA | 0 (0.0) | NA | NA | NA | NA | NA | NA |
| TT/AA | 14 (3.5) | 2 (1.2) | 0.297 (0.066 - 1.332) | 0.113 | 0.226 | 0.315 (0.068 - 1.462) | 0.14 | 0.28 | 2 (1.2) | 0.297 (0.066 - 1.332) | 0.113 | 0.226 | 0.315 (0.068 - 1.462) | 0.14 | 0.28 |
| TT/AC | 3 (0.8) | 0 (0.0) | NA | NA | NA | NA | NA | NA | 0 (0.0) | NA | NA | NA | NA | NA | NA |
| TT/CC | 0 (0.0) | 0 (0.0) | NA | NA | NA | NA | NA | NA | 0 (0.0) | NA | NA | NA | NA | NA | NA |

^a^Adjusted odds ratio on the basis of risk factors, such as age, gender, hypertension, diabetes mellitus.

^b^False positive discovery rate (FDR)-adjusted *P*-value.

**Table F in S1 File.** Genotype frequencies of miRNA biogenesis gene polymorphisms and CRC patients survival in TNM stage I + II

|  |  | Overall survival | | |  | Relapse-free survival* | | | |
| --- | --- | --- | --- | --- | --- | --- | --- | --- | --- |
| Genotypes | Patients (n=177) | Death (n=15) | Adjusted HR  (95% CI)^a^ | *P* | *FDR*^b^ | Relapse (n=11) | Adjusted HR  (95% CI)^a^ | *P* | *FDR*^b^ |
| ***DICER* rs3742330** |  |  |  |  |  |  |  |  |  |
| AA | 57 (32.2) | 3 (20.0) | 1.000 (reference) |  |  | 2 (18.2) | 1.000 (reference) |  |  |
| AG | 91 (51.4) | 9 (60.0) | 1.474 (0.368 - 5.899) | 0.586 | 0.586 | 6 (54.5) | 5.730 (0.623 - 52.709) | 0.125 | 0.310 |
| GG | 29 (16.4) | 3 (20.0) | 6.880 (0.128 - 370.765) | 0.346 | N/A | 3 (27.3) | 1.935 (0.110 - 34.071) | 0.654 | 0.654 |
| Dominant ( AA vs. AG + GG ) |  |  | 1.371 (0.372 - 5.055) | 0.638 | 0.704 |  | 4.998 (0.622 - 40.153) | 0.132 | 0.390 |
| Recessive ( AA + AG vs. GG ) |  |  | 1.426 (0.370 - 5.504) | 0.608 | N/A |  | 2.217 (0.561 - 8.763) | 0.259 | 0.389 |
|  |  |  |  |  |  |  |  |  |  |
| ***DROSHA* rs10719** |  |  |  |  |  |  |  |  |  |
| TT | 94 (53.1) | 6 (40.0) | 1.000 (reference) |  |  | 5 (45.5) | 1.000 (reference) |  |  |
| TC | 69 (39.0) | 9 (60.0) | 2.113 (0.700 - 6.380) | 0.187 | 0.374 | 5 (45.5) | 1.502 (0.396 - 5.690) | 0.552 | 0.616 |
| CC | 14 (7.9) | 0 (0.0) | N/A | N/A | N/A | 1 (9.1) | 2.203 (0.214 - 22.714) | 0.509 | 0.654 |
| Dominant ( TT vs. TC + CC ) |  |  | 1.821 (0.599 - 5.536) | 0.293 | 0.586 |  | 1.594 (0.445 - 5.712) | 0.476 | 0.476 |
| Recessive ( TT + TC vs. CC ) |  |  | N/A | N/A | N/A |  | 1.313 (0.156 - 11.035) | 0.803 | 0.803 |
|  |  |  |  |  |  |  |  |  |  |
| ***RAN* rs14035** |  |  |  |  |  |  |  |  |  |
| CC | 114 (64.4) | 7 (46.7) | 1.000 (reference) |  |  | 5 (45.5) | 1.000 (reference) |  |  |
| CT | 57 (32.2) | 8 (53.3) | 2.241 (0.714 - 7.031) | 0.169 | 0.374 | 5 (45.5) | 1.429 (0.356 - 5.741) | 0.616 | 0.616 |
| TT | 6 (3.4) | 0 (0.0) | N/A | N/A | N/A | 1 (9.1) | 2.939 (0.291 - 29.671) | 0.363 | 0.654 |
| Dominant ( CC vs. CT + TT ) |  |  | 2.083 (0.662 - 6.555) | 0.212 | 0.586 |  | 1.616 (0.435 - 6.006) | 0.476 | 0.476 |
| Recessive ( CC + CT vs. TT ) |  |  | N/A | N/A | N/A |  | 3.999 (0.447 - 35.741) | 0.217 | 0.389 |
|  |  |  |  |  |  |  |  |  |  |
| ***XPO5* rs11077** |  |  |  |  |  |  |  |  |  |
| AA | 145 (81.9) | 12 (80.0) | 1.000 (reference) |  |  | 8 (72.7) | 1.000 (reference) |  |  |
| AC | 31 (17.5) | 3 (20.0) | 1.487 (0.403 - 5.491) | 0.554 | 0.586 | 3 (27.3) | 2.825 (0.679 - 11.749) | 0.155 | 0.310 |
| CC | 1 (0.6) | 0 (0.0) | N/A | N/A | N/A | 0 (0.0) | N/A | N/A | N/A |
| Dominant ( AA vs. AC + CC ) |  |  | 1.287 (0.353 - 4.696) | 0.704 | 0.704 |  | 2.574 (0.620 - 10.689) | 0.195 | 0.390 |
| Recessive ( AA + AC vs. CC ) |  |  | N/A | N/A | N/A |  | N/A | N/A | N/A |

^a^Adjusted odds ratio on the basis of risk factors, such as age, gender, hypertension, diabetes mellitus.

^b^False positive discovery rate (FDR)-adjusted *P*-value. *Relapse-free survival that includes a group of patients who appear to have experienced a relapse.

**Table G in S1 File.** Genotype frequencies of miRNA biogenesis genes polymorphisms and CRC patients survival in TNM stage III + IV

|  |  | Overall survival | | |  | Relapse-free survival* | | | |
| --- | --- | --- | --- | --- | --- | --- | --- | --- | --- |
| Genotypes | Patients (n=163) | Death (n=63) | Adjusted HR  (95% CI)^a^ | *P* | *FDR*^b^ | Relapse (n=59) | Adjusted HR  (95% CI)^a^ | *P* | *FDR*^b^ |
| ***DICER* rs3742330** |  |  |  |  |  |  |  |  |  |
| AA | 47 (28.8) | 17 (27.0) | 1.000 (reference) |  |  | 18 (30.5) | 1.000 (reference) |  |  |
| AG | 84 (51.5) | 33 (52.4) | 0.965 (0.529 - 1.762) | 0.909 | 0.909 | 30 (50.8) | 0.889 (0.488 - 1.619) | 0.702 | 0.702 |
| GG | 32 (19.6) | 13 (20.6) | 1.809 (0.834 - 3.923) | 0.135 | 0.405 | 11 (18.6) | 1.349 (0.608 - 2.996) | 0.464 | 0.607 |
| Dominant ( AA vs. AG + GG ) |  |  | 1.085 (0.619 - 1.903) | 0.777 | 0.777 |  | 0.960 (0.548 - 1.682) | 0.888 | 0.888 |
| Recessive ( AA + AG vs. GG ) |  |  | 1.468 (0.777 - 2.773) | 0.239 | 0.717 |  | 1.239 (0.626 - 2.453) | 0.540 | 0.756 |
|  |  |  |  |  |  |  |  |  |  |
| ***DROSHA* rs10719** |  |  |  |  |  |  |  |  |  |
| TT | 89 (54.6) | 31 (49.2) | 1.000 (reference) |  |  | 32 (54.2) | 1.000 (reference) |  |  |
| TC | 63 (38.7) | 28 (44.4) | 1.627 (0.942 - 2.811) | 0.083 | 0.332 | 22 (37.3) | 1.346 (0.753 - 2.409) | 0.319 | 0.702 |
| CC | 11 (6.7) | 4 (6.3) | 1.480 (0.499 - 4.394) | 0.482 | 0.501 | 5 (8.5) | 2.043 (0.751 - 5.555) | 0.164 | 0.492 |
| Dominant ( TT vs. TC + CC ) |  |  | 1.571 (0.927 - 2.664) | 0.095 | 0.380 |  | 1.413 (0.814 - 2.454) | 0.222 | 0.709 |
| Recessive ( TT + TC vs. CC ) |  |  | 1.103 (0.395 - 3.080) | 0.852 | 0.852 |  | 1.640 (0.641 - 4.194) | 0.304 | 0.756 |
|  |  |  |  |  |  |  |  |  |  |
| ***RAN* rs14035** |  |  |  |  |  |  |  |  |  |
| CC | 108 (66.3) | 43 (68.3) | 1.000 (reference) |  |  | 41 (69.5) | 1.000 (reference) |  |  |
| CT | 50 (30.7) | 19 (30.2) | 0.851 (0.497 - 1.458) | 0.559 | 0.807 | 16 (27.1) | 0.792 (0.444 - 1.411) | 0.430 | 0.702 |
| TT | 5 (3.1) | 1 (1.6) | 0.502 (0.068 - 3.707) | 0.501 | 0.501 | 2 (3.4) | 1.477 (0.337 - 6.479) | 0.607 | 0.607 |
| Dominant ( CC vs. CT + TT ) |  |  | 0.822 (0.484 - 1.396) | 0.471 | 0.777 |  | 0.820 (0.471 - 1.430) | 0.487 | 0.709 |
| Recessive ( CC + CT vs. TT ) |  |  | 0.522 (0.072 - 3.798) | 0.523 | 0.785 |  | 1.258 (0.298 - 5.318) | 0.756 | 0.756 |
|  |  |  |  |  |  |  |  |  |  |
| ***XPO5* rs11077** |  |  |  |  |  |  |  |  |  |
| AA | 132 (81.0) | 50 (79.4) | 1.000 (reference) |  |  | 46 (78.0) | 1.000 (reference) |  |  |
| AC | 31 (19.0) | 13 (20.6) | 1.185 (0.625 - 2.250) | 0.605 | 0.807 | 13 (22.0) | 1.230 (0.644 - 2.350) | 0.532 | 0.702 |
| CC | 0 (0.0) | 0 (0.0) | N/A | N/A | N/A | 0 (0.0) | N/A | N/A | N/A |
| Dominant ( AA vs. AC + CC ) |  |  | 1.185 (0.625 - 2.250) | 0.605 | 0.777 |  | 1.230 (0.644 - 2.350) | 0.532 | 0.709 |
| Recessive ( AA + AC vs. CC ) |  |  | N/A | N/A | N/A |  | N/A | N/A | N/A |

^a^Adjusted odds ratio on the basis of risk factors, such as age, gender, hypertension, diabetes mellitus.

^b^False positive discovery rate (FDR)-adjusted *P*-value. *Relapse-free survival that includes a group of patients who appear to have experienced a relapse.

**Table H in S1 File.** Allele combinations of miRNA processing genes in CRC patients and controlsusing multifactor dimensionality reduction

| Characteristics | Overall | Control | Case | Control | Case | OR(95% CI)^a^ | *P* | *FDR*^b^ | OR(95% CI)^a^ | *P* | *FDR*^b^ |
| --- | --- | --- | --- | --- | --- | --- | --- | --- | --- | --- | --- |
| ***DICER*/*DROSHA*/*RAN*/*XPO5*** | | | | 800 | 816 |  |  |  |  |  |  |
| A-T-C-A | 0.6043 | 0.5986 | 0.6100 | 479 | 498 | 1.000 (reference) |  |  | 1.019 (0.8695 - 1.195) | 0.845 | 1.000 |
| A-T-C-C | 0.0099 | 0.0106 | 0.0093 | 8 | 8 | 0.962 (0.358 - 2.580) | 1.000 | 1.000 | 0.980 (0.3661 - 2.625) | 1.000 | 1.000 |
| A-T-T-A | 0.1046 | 0.1150 | 0.0944 | 92 | 77 | 0.805 (0.580 - 1.117) | 0.212 | 1.000 | 0.821 (0.5970 - 1.128) | 0.226 | 1.000 |
| A-C-C-A | 0.0587 | 0.0619 | 0.0555 | 50 | 45 | 0.866 (0.568 - 1.32) | 0.521 | 1.000 | 0.882 (0.5830 - 1.336) | 0.598 | 1.000 |
| A-C-C-C | 0.0096 | 0.0083 | 0.0109 | 7 | 9 | 1.237 (0.4568 - 3.348) | 0.803 | 1.000 | 1.261 (0.4671 - 3.402) | 0.803 | 1.000 |
| G-T-C-A | 0.1344 | 0.1315 | 0.1371 | 105 | 112 | 1.026 (0.7643 - 1.377) | 0.881 | 1.000 | 1.046 (0.7875 - 1.389) | 0.773 | 1.000 |
| G-T-C-C | 0.0156 | 0.0130 | 0.0181 | 10 | 15 | 1.443 (0.6417 - 3.244) | 0.422 | 1.000 | 1.471 (0.6566 - 3.293) | 0.422 | 1.000 |
| G-C-C-A | 0.0544 | 0.0524 | 0.0564 | 42 | 46 | 1.053 (0.6806 - 1.630) | 0.825 | 1.000 | 1.074 (0.6988 - 1.650) | 0.827 | 1.000 |
| G-C-C-C | 0.0085 | 0.0087 | 0.0083 | 7 | 7 | 0.9618 (0.3348 - 2.763) | 1.000 | 1.000 | 0.980 (0.3422 - 2.808) | 1.000 | 1.000 |
|  |  |  |  |  |  |  |  |  |  |  |  |
| ***DICER*/*DROSHA*/*RAN*** | | | |  |  |  |  |  |  |  |  |
| A-T-C | 0.6139 | 0.6090 | 0.6188 | 487 | 505 | 1.000 (reference) |  |  | 1.017 (0.8679 - 1.191) | 0.870 | 0.922 |
| A-T-T | 0.1046 | 0.1150 | 0.0944 | 92 | 77 | 0.8071 (0.5818 - 1.120) | 0.212 | 0.8492 | 0.821 (0.5970 - 1.128) | 0.226 | 0.922 |
| A-C-T | 0.0686 | 0.0703 | 0.0669 | 56 | 55 | 0.9471 (0.6397 - 1.402) | 0.842 | 0.917 | 0.963 (0.6554 - 1.415) | 0.922 | 0.922 |
| G-T-C | 0.1503 | 0.1447 | 0.1557 | 116 | 127 | 1.056 (0.7973 - 1.398) | 0.721 | 0.917 | 1.073 (0.8194 - 1.406) | 0.630 | 0.922 |
| G-C-T | 0.0626 | 0.0609 | 0.0642 | 49 | 52 | 1.023 (0.6794 - 1.542) | 0.917 | 0.917 | 1.040 (0.6958 - 1.556) | 0.918 | 0.922 |
|  |  |  |  |  |  |  |  |  |  |  |  |
| ***DICER*/*DROSHA*/*XPO5*** | | | |  |  |  |  |  |  |  |  |
| A-T-A | 0.7113 | 0.7161 | 0.7066 | 573 | 577 | 1.000 (reference) |  |  | 0.987 (0.8486 - 1.148) | 0.898 | 1.000 |
| A-T-C | 0.0095 | 0.0100 | 0.0090 | 8 | 7 | 0.8689 (0.3130 - 2.413) | 0.802 | 0.936 | 0.858 (0.3095 - 2.377) | 0.801 | 1.000 |
| A-C-A | 0.0569 | 0.0600 | 0.0537 | 48 | 44 | 0.9103 (0.5950 - 1.393) | 0.745 | 0.936 | 0.899 (0.5901 - 1.369) | 0.669 | 1.000 |
| A-C-C | 0.0095 | 0.0083 | 0.0107 | 7 | 9 | 1.277 (0.4722 - 3.452) | 0.802 | 0.936 | 1.261 (0.4671 - 3.402) | 0.803 | 1.000 |
| G-T-A | 0.1323 | 0.1295 | 0.1351 | 104 | 110 | 1.05 (0.7844 - 1.407) | 0.766 | 0.936 | 1.037 (0.7796 - 1.379) | 0.827 | 1.000 |
| G-T-C | 0.0157 | 0.0132 | 0.0182 | 11 | 15 | 1.354 (0.6166 - 2.974) | 0.553 | 0.936 | 1.337 (0.6102 - 2.929) | 0.555 | 1.000 |
| G-C-A | 0.0559 | 0.0538 | 0.0580 | 43 | 47 | 1.085 (0.7064 - 1.668) | 0.743 | 0.936 | 1.072 (0.7005 - 1.639) | 0.829 | 1.000 |
| G-C-C | 0.0089 | 0.0091 | 0.0087 | 7 | 7 | 0.9931 (0.3460 - 2.850) | 1.000 | 1.000 | 0.980 (0.3422 - 2.808) | 1.000 | 1.000 |
|  |  |  |  |  |  |  |  |  |  |  |  |
| ***DICER*/ *RAN*/*XPO5*** | | | |  |  |  |  |  |  |  |  |
| A-C-A | 0.6623 | 0.6599 | 0.6646 | 528 | 542 | 1.000 (reference) |  |  | 1.006 (0.8623 - 1.175) | 0.967 | 1.000 |
| A-C-C | 0.0203 | 0.0195 | 0.0210 | 16 | 17 | 1.035 (0.5175 - 2.070) | 1.000 | 1.000 | 1.042 (0.5226 - 2.076) | 1.000 | 1.000 |
| A-T-A | 0.1046 | 0.1150 | 0.0944 | 92 | 77 | 0.8153 (0.5887 - 1.129) | 0.247 | 0.986 | 0.821 (0.5970 - 1.128) | 0.226 | 1.000 |
| G-C-A | 0.1895 | 0.1845 | 0.1944 | 148 | 159 | 1.047 (0.8118 - 1.349) | 0.746 | 0.995 | 1.053 (0.8250 - 1.345) | 0.709 | 1.000 |
| G-C-C | 0.0234 | 0.0211 | 0.0255 | 17 | 21 | 1.203 (0.6278 - 2.307) | 0.623 | 0.995 | 1.211 (0.6341 - 2.313) | 0.624 | 1.000 |
|  |  |  |  |  |  |  |  |  |  |  |  |
| ***DROSHA*/*RAN*/*XPO5*** | | | |  |  |  |  |  |  |  |  |
| T-C-A | 0.7379 | 0.7294 | 0.7461 | 584 | 609 | 1.000 (reference) |  |  | 1.022 (0.8802 - 1.187) | 0.802 | 0.938 |
| T-C-C | 0.0264 | 0.0243 | 0.0284 | 19 | 23 | 1.161 (0.6255 - 2.154) | 0.754 | 0.850 | 1.187 (0.6413 - 2.196) | 0.641 | 0.938 |
| T-T-A | 0.1046 | 0.1150 | 0.0944 | 92 | 77 | 0.8026 (0.5808 - 1.109) | 0.189 | 0.757 | 0.821 (0.5970 - 1.128) | 0.226 | 0.938 |
| C-C-A | 0.1139 | 0.1149 | 0.1129 | 92 | 92 | 0.9589 (0.7029 - 1.308) | 0.813 | 0.850 | 0.981 (0.7226 - 1.330) | 0.938 | 0.938 |
| C-C-C | 0.0173 | 0.0163 | 0.0182 | 13 | 15 | 1.106 (0.5219 - 2.346) | 0.850 | 0.850 | 1.131 (0.5348 - 2.393) | 0.850 | 0.938 |
|  |  |  |  |  |  |  |  |  |  |  |  |
| ***DICER*/*DROSHA*** | | | |  |  |  |  |  |  |  |  |
| A-T | 0.7205 | 0.7259 | 0.7153 | 581 | 584 | 1.000 (reference) |  |  | 0.986 (0.8476 - 1.146) | 0.879 | 0.879 |
| A-C | 0.0666 | 0.0685 | 0.0648 | 55 | 53 | 0.9587 (0.6462 - 1.422) | 0.841 | 0.841 | 0.945 (0.6398 - 1.395) | 0.843 | 0.879 |
| G-T | 0.1483 | 0.1429 | 0.1536 | 114 | 125 | 1.091 (0.8256 - 1.441) | 0.570 | 0.841 | 1.075 (0.8190 - 1.411) | 0.628 | 0.879 |
| G-C | 0.0646 | 0.0628 | 0.0664 | 50 | 54 | 1.074 (0.7191 - 1.605) | 0.759 | 0.841 | 1.059 (0.7119 - 1.575) | 0.840 | 0.879 |
|  |  |  |  |  |  |  |  |  |  |  |  |
| ***DICER*/*RAN*7** | | | |  |  |  |  |  |  |  |  |
| A-C | 0.6825 | 0.6794 | 0.6857 | 544 | 560 | 1.000 (reference) |  |  | 1.009 (0.8660 - 1.176) | 0.937 | 0.937 |
| A-T | 0.1046 | 0.1150 | 0.0944 | 92 | 77 | 0.813 (0.5875 - 1.125) | 0.216 | 0.4328 | 0.821 (0.5970 - 1.128) | 0.226 | 0.679 |
| G-C | 0.2129 | 0.2056 | 0.2200 | 164 | 180 | 1.066 (0.8368 - 1.359) | 0.622 | 0.6215 | 1.076 (0.8524 - 1.358) | 0.553 | 0.829 |
|  |  |  |  |  |  |  |  |  |  |  |  |
| ***DICER*/*XPO5*** | | | |  |  |  |  |  |  |  |  |
| A-A | 0.7674 | 0.7755 | 0.7595 | 620 | 620 | 1.000 (reference) |  |  | 0.98 (0.8455 - 1.137) | 0.822 | 0.859 |
| A-C | 0.0197 | 0.0188 | 0.0206 | 15 | 17 | 1.133 (0.5609 - 2.290) | 0.858 | 0.858 | 1.111 (0.5510 - 2.240) | 0.859 | 0.859 |
| G-A | 0.1889 | 0.1838 | 0.1940 | 147 | 158 | 1.075 (0.8365 - 1.381) | 0.609 | 0.858 | 1.054 (0.8248 - 1.346) | 0.708 | 0.859 |
| G-C | 0.0239 | 0.0218 | 0.0260 | 17 | 21 | 1.235 (0.6454 - 2.364) | 0.622 | 0.858 | 1.211 (0.6341 - 2.313) | 0.624 | 0.859 |
|  |  |  |  |  |  |  |  |  |  |  |  |
| ***DROSHA*/*RAN*** | | | |  |  |  |  |  |  |  |  |
| T-C | 0.7642 | 0.7538 | 0.7745 | 603 | 632 | 1.000 (reference) |  |  | 1.028 (0.8860 - 1.192) | 0.748 | 1.000 |
| T-T | 0.1046 | 0.1150 | 0.0944 | 92 | 77 | 0.7986 (0.5783 - 1.103) | 0.189 | 0.379 | 0.821 (0.5970 - 1.128) | 0.226 | 0.679 |
| C-C | 0.1312 | 0.1312 | 0.1311 | 105 | 107 | 0.9723 (0.7264 - 1.301) | 0.882 | 0.882 | 0.999 (0.7502 - 1.330) | 1.000 | 1.000 |
|  |  |  |  |  |  |  |  |  |  |  |  |
| ***DROSHA*//*XPO5*** | | | |  |  |  |  |  |  |  |  |
| T-A | 0.8427 | 0.8449 | 0.8406 | 676 | 686 | 1.000 (reference) |  |  | 0.995 (0.8613 - 1.149) | 0.974 | 0.974 |
| T-C | 0.0261 | 0.0239 | 0.0283 | 19 | 23 | 1.193 (0.6436 - 2.211) | 0.640 | 0.938 | 1.187 (0.6413 - 2.196) | 0.641 | 0.974 |
| C-A | 0.1137 | 0.1145 | 0.1129 | 92 | 92 | 0.9854 (0.7242 - 1.341) | 0.938 | 0.938 | 0.980 (0.7226 - 1.330) | 0.938 | 0.974 |
| C-C | 0.0175 | 0.0168 | 0.0183 | 13 | 15 | 1.137 (0.5369 - 2.408) | 0.849 | 0.938 | 1.131 (0.5348 - 2.393) | 0.850 | 0.974 |
|  |  |  |  |  |  |  |  |  |  |  |  |
| ***RAN*/*XPO5*** | | | |  |  |  |  |  |  |  |  |
| C-A | 0.8518 | 0.8444 | 0.8591 | 676 | 701 | 1.000 (reference) |  |  | 1.017 (0.8805 - 1.174) | 0.851 | 0.851 |
| C-C | 0.0436 | 0.0406 | 0.0466 | 32 | 38 | 1.145 (0.7071 - 1.855) | 0.625 | 0.625 | 1.164 (0.7201 - 1.882) | 0.544 | 0.817 |
| T-A | 0.1046 | 0.1150 | 0.0944 | 92 | 77 | 0.8071 (0.5857 - 1.112) | 0.193 | 0.387 | 0.821 (0.5970 - 1.128) | 0.226 | 0.679 |

^a^Adjusted odds ratio on the basis of risk factors, such as age, gender, hypertension, diabetes mellitus.

^b^False positive discovery rate (FDR)-adjusted *P*-value.

| **Table I in S1 File.** Allele combination of miRNA biogenesis genes polymorphisms based on multifactor dimensionality reduction and CRC patients survival | | | | | | | | | | | | | |
| --- | --- | --- | --- | --- | --- | --- | --- | --- | --- | --- | --- | --- | --- |
|  |  |  |  |  |  |  | Overall survival | |  |  | Relapse-free survival* | | |
| Characteristics | Overall | Control | Case | Control | Case | Death | Adjusted HR  (95% CI)^a^ | *P* | *FDR*^b^ | Relapse | Adjusted HR  (95% CI)^a^ | *P* | *FDR*^b^ |
| ***DICER*/*DROSHA*/*RAN*/*XPO5* (rs3742330,rs10719,rs14035,rs11077)** | | | | 800 | 816 | n=131 |  |  |  | n=117 |  |  |  |
| A-T-C-A | 0.6043 | 0.5986 | 0.6100 | 479 | 498 | 63 (48.1) | 1.000 (reference) |  |  | 55 (47.0) | 1.000 (reference) |  |  |
| A-T-C-C | 0.0099 | 0.0106 | 0.0093 | 8 | 8 | 0 (0.0) | N/A | 0.95 | 0.982 | 0 (0.0) | N/A | 0.982 | 0.982 |
| A-T-T-A | 0.1046 | 0.1150 | 0.0944 | 92 | 77 | 4 (3.1) | 0.987 (0.350 - 2.784) | 0.98 | 0.982 | 4 (3.4) | 0.585 (0.170 - 2.007) | 0.396 | 0.861 |
| A-C-C-A | 0.0587 | 0.0619 | 0.0555 | 50 | 45 | 11 (8.4) | 1.458 (0.724 - 2.938) | 0.294 | 0.982 | 11 (9.4) | 1.705 (0.864 - 3.365) | 0.126 | 0.861 |
| A-C-C-C | 0.0096 | 0.0083 | 0.0109 | 7 | 9 | 1 (0.8) | 0.690 (0.095 - 4.985) | 0.714 | 0.982 | 1 (0.9) | 0.663 (0.092 - 4.779) | 0.685 | 0.861 |
| G-T-C-A | 0.1344 | 0.1315 | 0.1371 | 105 | 112 | 27 (20.6) | 0.994 (0.591 - 1.672) | 0.982 | 0.982 | 25 (21.4) | 1.091 (0.638 - 1.865) | 0.753 | 0.861 |
| G-T-C-C | 0.0156 | 0.0130 | 0.0181 | 10 | 15 | 5 (3.8) | 1.642 (0.627 - 4.300) | 0.315 | 0.982 | 6 (5.1) | 1.745 (0.719 - 4.233) | 0.221 | 0.861 |
| G-C-C-A | 0.0544 | 0.0524 | 0.0564 | 42 | 46 | 18 (13.7) | 1.148 (0.661 - 1.993) | 0.626 | 0.982 | 13 (11.1) | 1.190 (0.622 - 2.276) | 0.601 | 0.861 |
| G-C-C-C | 0.0085 | 0.0087 | 0.0083 | 7 | 7 | 2 (1.5) | 1.308 (0.310 - 5.529) | 0.716 | 0.982 | 2 (1.7) | 1.698 (0.393 - 7.339) | 0.481 | 0.861 |
|  |  |  |  |  |  |  |  |  |  |  |  |  |  |
| ***DICER*/*DROSHA*/*RAN* (rs3742330, rs10719, rs14035)** | | | |  |  | n=114 |  |  |  | n=103 |  |  |  |
| A-T-C | 0.6139 | 0.6090 | 0.6188 | 487 | 505 | 63 (55.3) | 1.000 (reference) |  |  | 55 (53.4) | 1.000 (reference) |  |  |
| A-T-T | 0.1046 | 0.1150 | 0.0944 | 92 | 77 | 6 (5.3) | 1.335 (0.560 - 3.182) | 0.517 | 0.682 | 5 (4.9) | 0.797 (0.268 - 2.369) | 0.684 | 0.684 |
| A-C-T | 0.0686 | 0.0703 | 0.0669 | 56 | 55 | 1 (0.9) | 0.401 (0.056 - 2.902) | 0.368 | 0.682 | 4 (3.9) | 1.457 (0.445 - 4.769) | 0.536 | 0.684 |
| G-T-C | 0.1503 | 0.1447 | 0.1557 | 116 | 127 | 32 (28.1) | 1.107 (0.682 - 1.797) | 0.682 | 0.682 | 31 (30.1) | 1.211 (0.739 - 1.984) | 0.449 | 0.684 |
| G-C-T | 0.0626 | 0.0609 | 0.0642 | 49 | 52 | 12 (10.5) | 1.779 (0.906 - 3.494) | 0.096 | 0.384 | 8 (7.8) | 1.597 (0.694 - 3.676) | 0.273 | 0.684 |
|  |  |  |  |  |  |  |  |  |  |  |  |  |  |
| ***DICER*/*DROSHA*/*XPO5* (rs3742330, rs10719, rs11077)** | | | |  |  | n=156 |  |  |  | n=140 |  |  |  |
| A-T-A | 0.7113 | 0.7161 | 0.7066 | 573 | 577 | 67 (42.9) | 1.000 (reference) |  |  | 59 (42.1) | 1.000 (reference) |  |  |
| A-T-C | 0.0095 | 0.0100 | 0.0090 | 8 | 7 | 2 (1.3) | 1.025 (0.245 - 4.286) | 0.974 | 0.974 | 1 (0.7) | 0.939 (0.127 - 6.941) | 0.951 | 0.951 |
| A-C-A | 0.0569 | 0.0600 | 0.0537 | 48 | 44 | 11 (7.1) | 1.228 (0.616 - 2.448) | 0.562 | 0.974 | 13 (9.3) | 1.609 (0.839 - 3.086) | 0.155 | 0.791 |
| A-C-C | 0.0095 | 0.0083 | 0.0107 | 7 | 9 | 2 (1.3) | 0.756 (0.185 - 3.094) | 0.698 | 0.974 | 3 (2.1) | 1.361 (0.423 - 4.379) | 0.607 | 0.792 |
| G-T-A | 0.1323 | 0.1295 | 0.1351 | 104 | 110 | 35 (22.4) | 0.965 (0.605 - 1.540) | 0.883 | 0.974 | 33 (23.6) | 1.207 (0.741 - 1.965) | 0.452 | 0.791 |
| G-T-C | 0.0157 | 0.0132 | 0.0182 | 11 | 15 | 7 (4.5) | 1.178 (0.525 - 2.644) | 0.693 | 0.974 | 8 (5.7) | 1.179 (0.543 - 2.558) | 0.679 | 0.792 |
| G-C-A | 0.0559 | 0.0538 | 0.0580 | 43 | 47 | 27 (17.3) | 1.204 (0.749 - 1.935) | 0.447 | 0.974 | 19 (13.6) | 1.279 (0.736 - 2.223) | 0.385 | 0.791 |
| G-C-C | 0.0089 | 0.0091 | 0.0087 | 7 | 7 | 5 (3.2) | 2.015 (0.780 - 5.206) | 0.15 | 0.974 | 4 (2.9) | 1.999 (0.588 - 6.791) | 0.269 | 0.791 |
|  |  |  |  |  |  |  |  |  |  |  |  |  |  |
| ***DICER*/*RAN*/*XPO5* (rs3742330, rs14035, rs11077)** | | | |  |  | n=131 |  |  |  | n=119 |  |  |  |
| A-C-A | 0.6623 | 0.6599 | 0.6646 | 528 | 542 | 74 (56.5) | 1.000 (reference) |  |  | 66 (55.5) | 1.000 (reference) |  |  |
| A-C-C | 0.0203 | 0.0195 | 0.0210 | 16 | 17 | 1 (0.8) | 0.322 (0.045 - 2.333) | 0.265 | 0.704 | 1 (0.8) | 0.368 (0.051 - 2.661) | 0.325 | 0.443 |
| A-T-A | 0.1046 | 0.1150 | 0.0944 | 92 | 77 | 4 (3.1) | 0.717 (0.256 - 2.005) | 0.528 | 0.704 | 6 (5.0) | 0.591 (0.206 - 1.700) | 0.332 | 0.443 |
| G-C-A | 0.1895 | 0.1845 | 0.1944 | 148 | 159 | 45 (34.4) | 0.953 (0.638 - 1.423) | 0.814 | 0.814 | 38 (31.9) | 0.967 (0.628 - 1.488) | 0.878 | 0.878 |
| G-C-C | 0.0234 | 0.0211 | 0.0255 | 17 | 21 | 7 (5.3) | 1.391 (0.632 - 3.062) | 0.415 | 0.704 | 8 (6.7) | 1.587 (0.751 - 3.353) | 0.229 | 0.443 |
|  |  |  |  |  |  |  |  |  |  |  |  |  |  |
| ***DROSHA*/*RAN*/XP*O5* (rs10719, rs14035, rs11077)** | | | |  |  | n=139 |  |  |  | n=125 |  |  |  |
| T-C-A | 0.7379 | 0.7294 | 0.7461 | 584 | 609 | 90 (64.7) | 1.000 (reference) |  |  | 80 (64.0) | 1.000 (reference) |  |  |
| T-C-C | 0.0264 | 0.0243 | 0.0284 | 19 | 23 | 5 (3.6) | 1.138 (0.449 - 2.884) | 0.787 | 0.928 | 6 (4.8) | 1.346 (0.574 - 3.158) | 0.497 | 0.917 |
| T-T-A | 0.1046 | 0.1150 | 0.0944 | 92 | 77 | 12 (8.6) | 0.897 (0.461 - 1.748) | 0.751 | 0.928 | 12 (9.6) | 0.909 (0.443 - 1.865) | 0.795 | 0.917 |
| C-C-A | 0.1139 | 0.1149 | 0.1129 | 92 | 92 | 29 (20.9) | 1.241 (0.792 - 1.943) | 0.348 | 0.928 | 24 (19.2) | 1.312 (0.808 - 2.130) | 0.275 | 0.917 |
| C-C-C | 0.0173 | 0.0163 | 0.0182 | 13 | 15 | 3 (2.2) | 1.055 (0.332 - 3.358) | 0.928 | 0.928 | 3 (2.4) | 1.064 (0.334 - 3.389) | 0.917 | 0.917 |
|  |  |  |  |  |  |  |  |  |  |  |  |  |  |
| ***DICER*/*DROSHA* (rs3742330, rs10719)** | | | |  |  | n=156 |  |  |  | n=140 |  |  |  |
| A-T | 0.7205 | 0.7259 | 0.7153 | 581 | 584 | 69 (44.2) | 1.000 (reference) |  |  | 60 (42.9) | 1.000 (reference) |  |  |
| A-C | 0.0666 | 0.0685 | 0.0648 | 55 | 53 | 13 (8.3) | 1.111 (0.590 - 2.093) | 0.745 | 0.896 | 16 (11.4) | 1.571 (0.868 - 2.841) | 0.137 | 0.399 |
| G-T | 0.1483 | 0.1429 | 0.1536 | 114 | 125 | 42 (26.9) | 1.029 (0.670 - 1.581) | 0.896 | 0.896 | 41 (29.3) | 1.175 (0.757 - 1.824) | 0.475 | 0.475 |
| G-C | 0.0646 | 0.0628 | 0.0664 | 50 | 54 | 32 (20.5) | 1.280 (0.819 - 1.999) | 0.281 | 0.843 | 23 (16.4) | 1.350 (0.798 - 2.285) | 0.266 | 0.399 |
|  |  |  |  |  |  |  |  |  |  |  |  |  |  |
| ***DICER*/*RAN* (rs3742330, rs14035)** | | | |  |  | n=134 |  |  |  | n=122 |  |  |  |
| A-C | 0.6825 | 0.6794 | 0.6857 | 544 | 560 | 75 (56.0) | 1.000 (reference) |  |  | 67 (54.9) | 1.000 (reference) |  |  |
| A-T | 0.1046 | 0.1150 | 0.0944 | 92 | 77 | 7 (5.2) | 0.972 (0.438 - 2.154) | 0.944 | 0.944 | 9 (7.4) | 0.962 (0.422 - 2.190) | 0.926 | 0.926 |
| G-C | 0.2129 | 0.2056 | 0.2200 | 164 | 180 | 52 (38.8) | 1.029 (0.705 - 1.504) | 0.882 | 0.944 | 46 (37.7) | 1.071 (0.718 - 1.600) | 0.737 | 0.926 |
|  |  |  |  |  |  |  |  |  |  |  |  |  |  |
| ***DICER*/*XPO5* (rs3742330, rs11077)** | | | |  |  | n=156 |  |  |  | n=140 |  |  |  |
| A-A | 0.7674 | 0.7755 | 0.7595 | 620 | 620 | 78 (50.0) | 1.000 (reference) |  |  | 72 (51.4) | 1.000 (reference) |  |  |
| A-C | 0.0197 | 0.0188 | 0.0206 | 15 | 17 | 4 (2.6) | 0.842 (0.305 - 2.326) | 0.741 | 0.998 | 4 (2.9) | 1.110 (0.400 - 3.080) | 0.841 | 0.841 |
| G-A | 0.1889 | 0.1838 | 0.1940 | 147 | 158 | 62 (39.7) | 1.000 (0.697 - 1.433) | 0.998 | 0.998 | 52 (37.1) | 1.082 (0.735 - 1.594) | 0.69 | 0.841 |
| G-C | 0.0239 | 0.0218 | 0.0260 | 17 | 21 | 12 (7.7) | 1.329 (0.712 - 2.479) | 0.374 | 0.998 | 12 (8.6) | 1.212 (0.629 - 2.337) | 0.567 | 0.841 |
|  |  |  |  |  |  |  |  |  |  |  |  |  |  |
| ***DROSHA*/*RAN* ( rs10719, rs14035)** | | | |  |  | n=143 |  |  |  | n=128 |  |  |  |
| T-C | 0.7642 | 0.7538 | 0.7745 | 603 | 632 | 95 (66.4) | 1.000 (reference) |  |  | 86 (67.2) | 1.000 (reference) |  |  |
| T-T | 0.1046 | 0.1150 | 0.0944 | 92 | 77 | 16 (11.2) | 0.982 (0.554 - 1.742) | 0.952 | 0.952 | 15 (11.7) | 0.869 (0.460 - 1.642) | 0.667 | 0.667 |
| C-C | 0.1312 | 0.1312 | 0.1311 | 105 | 107 | 32 (22.4) | 1.205 (0.785 - 1.850) | 0.396 | 0.792 | 27 (21.1) | 1.271 (0.803 - 2.013) | 0.309 | 0.618 |
|  |  |  |  |  |  |  |  |  |  |  |  |  |  |
| ***DROSHA*/*XPO5* ( rs10719, rs11077)** | | | |  |  | n=156 |  |  |  | n=140 |  |  |  |
| T-A | 0.8427 | 0.8449 | 0.8406 | 676 | 686 | 102 (65.4) | 1.000 (reference) |  |  | 92 (65.7) | 1.000 (reference) |  |  |
| T-C | 0.0261 | 0.0239 | 0.0283 | 19 | 23 | 9 (5.8) | 1.209 (0.599 - 2.440) | 0.598 | 0.598 | 9 (6.4) | 1.086 (0.534 - 2.209) | 0.822 | 0.822 |
| C-A | 0.1137 | 0.1145 | 0.1129 | 92 | 92 | 38 (24.4) | 1.213 (0.814 - 1.807) | 0.345 | 0.598 | 32 (22.9) | 1.265 (0.823 - 1.945) | 0.286 | 0.617 |
| C-C | 0.0175 | 0.0168 | 0.0183 | 13 | 15 | 7 (4.5) | 1.398 (0.643 - 3.039) | 0.401 | 0.598 | 7 (5.0) | 1.425 (0.615 - 3.305) | 0.411 | 0.617 |
|  |  |  |  |  |  |  |  |  |  |  |  |  |  |
| ***RAN*/*XPO5* (rs14035, rs11077)** | | | |  |  | n=135 |  |  |  | n=133 |  |  |  |
| C-A | 0.8518 | 0.8444 | 0.8591 | 676 | 701 | 119 (80.4) | 1.000 (reference) |  |  | 104 (78.2) | 1.000 (reference) |  |  |
| C-C | 0.0436 | 0.0406 | 0.0466 | 32 | 38 | 8 (5.4) | 0.981 (0.475 - 2.027) | 0.96 | 0.960 | 9 (6.8) | 1.150 (0.578 - 2.289) | 0.693 | 0.986 |
| T-A | 0.1046 | 0.1150 | 0.0944 | 92 | 77 | 21 (14.2) | 0.941 (0.567 - 1.562) | 0.815 | 0.960 | 20 (15.0) | 1.005 (0.583 - 1.731) | 0.986 | 0.986 |

^a^Adjusted odds ratio on the basis of risk factors, such as age, gender, hypertension, diabetes mellitus.

^b^False positive discovery rate (FDR)-adjusted *P*-value. ^*^Relapse-free survival that includes a group of patients who appear to have experienced a relapse.
